# Boron-Functionalized Graphitic Carbon Nitride Materials for Photocatalytic Applications: Effects on Chemical, Adsorptive, Optoelectronic, and Photocatalytic Properties

**DOI:** 10.1021/acsmaterialsau.5c00007

**Published:** 2025-05-12

**Authors:** Ioanna Itskou, Sharminaz C. Sageer, Daniel M. Dawson, Andreas Kafizas, Irena Nevjestic, Catriona M. McGilvery, Matyas Daboczi, Gwilherm Kerherve, Salvador Eslava, Sandrine Heutz, Sharon E. Ashbrook, Camille Petit

**Affiliations:** † Barrer Centre, Department of Chemical Engineering, 4615 Imperial College London, London SW7 2AZ, U.K.; ‡ School of Chemistry, EaStCHEM and Centre of Magnetic Resonance, 7486 University of St. Andrews, St. Andrews KY16 9ST, U.K.; § Department of Chemistry, Molecular Sciences Research Hub, 4615 Imperial College London, London W12 7TA, U.K.; ∥ London Centre for Nanotechnology, 4615 Imperial College London, London SW7 2AZ, U.K.; ⊥ Department of Materials, 4615 Imperial College London, London SW7 2AZ, U.K.; # Department of Chemical Engineering and Centre for Processable Electronics, 4615 Imperial College London, London SW7 2AZ, U.K.

**Keywords:** graphitic carbon nitride, boron, NMR spectroscopy, XPS, photocatalysis, NO_*x*_, CO_2_

## Abstract

Graphitic carbon nitride (gC_3_N_4_, or CN herein) is widely studied as a photocatalyst owing to its ease of synthesis, high stability, and optoelectronic properties. However, its photocatalytic performance often remains limited, and a common approach to tune its function and enhance its performance is by doping. Boron (B) functionalization of CN has showed a potential benefit on photocatalytic performance for several reactions. However, the reason for this improvement and the links between synthesis method, exact B chemical environment, and performance remain unclear. Here, we present a fundamental study that elucidates the influence of (i) B functionalization, (ii) B content, and (iii) choice of B precursor on the physicochemical, adsorptive, optoelectronic, and photocatalytic properties of bulk B-CN. We synthesized two sets of B-CN materials (0.5–11 at% B), using either elemental boron or boric acid as precursors. The samples were characterized using several imaging and spectroscopic techniques, which confirm the integration of B into the material through B–O bonding and the creation of B clusters in the case of the boron precursor, with density functional theory (DFT) calculations supporting our analyses. The distribution of B atoms within B-CN particles remained heterogeneous. Compared to CN, B-functionalized materials show enhanced porosity and CO_2_ uptake, with similar degrees of light absorption and deeper energy band positions. Transient absorption spectroscopy (TAS) measurements showed that charge carrier populations, lifetimes, and kinetics were not significantly affected by B functionalization; however, at 5 at% B doping, an increase in the concentration of charge carriers was seen. Higher B content enhances the photocatalytic NO_*x*_ removal under UVA irradiation (almost two-fold) and the selectivity to NO_3_^–^ from NO_*x*_ photooxidation, but has no significant effect on CO_2_ photoreduction, compared to pristine CN. Overall, this study provides fundamental insights to build on and more rationally produce better-performing B-CN photocatalysts.

## Graphical abstract

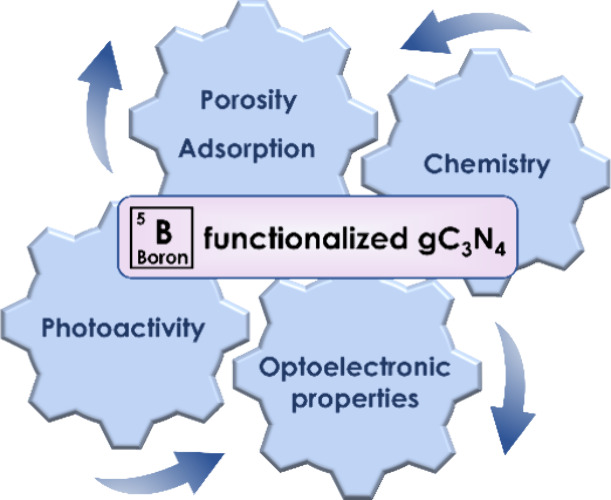

## 1 Introduction

Graphitic carbon nitride (gC_3_N_4_, or CN herein) is a nontoxic, easy to synthesize material comprising earth-abundant, nonmetallic elements, with high chemical and thermal stability and mechanical strength. Owing to these properties, CN has attracted much interest in recent years, and has been employed in various biomedical (e.g.*,* drug delivery, bioimaging), sensing (e.g*.,* bio- or gas sensing), energy storage (e.g*.,* supercapacitors, batteries, fuel cells), optical (e.g., light-emitting diodes, LEDs), and catalytic (e.g*.,* H_2_O splitting, CO_2_ reduction, air purification, N_2_ fixation) applications.^1^ In the context of catalysis, CN has been predominantly explored as a heterogeneous catalyst for thermal catalysis, electrocatalysis, and photocatalysis, with a strong focus on the latter.^2^ Such a strong focus can be explained by (i) the promising photocatalytic activity seen for a wide range of environmental purification and chemical/fuel production reactions and (ii) the unique optoelectronic properties of the material. Depending on the desired functionality, CN can be shaped into different forms by changing the synthetic route or adding treatment stages.^3^ A change in morphology usually results in a change in porosity and thereby a change in accessibility to active sites for reactant adsorption and reaction. Other than altering the morphology, one can also engineer the chemistry by heteroatom doping and the creation of defects, or the interfacial structure by the creation of heterojunctions and the use of cocatalysts.^3^ Such changes can influence a range of material properties, from porosity to optoelectronic properties to (photo)­catalytic activity. Indeed, CN is rarely used as a standalone catalyst; rather, it is often doped with nonmetals (e.g.*,* B, P, and S) or metal cocatalysts (e.g*.,* Co, Mo, Pt, Pd, and Au), purposely rendered defective (e.g.*,* O-rich, N-rich, V_O_, and V_N_), incorporated into heterojunctions (e.g*.,* CN/TiO_2_, CN/MOF), or a combination thereof.^1,2^

Investigation of boron-functionalized CN (herein referred to as B-CN) has recently intensified, and photocatalysts and photocatalytic systems incorporating B-CN have been studied for H_2_ production and O_2_ evolution,^4–13^ as well as for photodegradation of dyes and other organics.^14–24^ In both types of reactions, B-functionalized materials exhibited higher activity than pristine CN, and their superior performance was assigned to a combination of extended visible light absorption, better charge carrier separation, and easier access to surface redox sites. One study pointed to the potential bifunctional effect of elemental B when used to functionalize CN, by (i) efficiently separating and transporting charge carriers and (ii) converting light to thermal energy (photothermal effect), thus boosting photodegradation of sulfamerazine.^25^ A few research groups have tested B-CN-based materials for CO_2_ photoreduction,^24,26–31^ reporting higher CO (1.2–40.4 μmol g^–1^ h^–1^), CH_4_ (0.3–42.5 μmol g^–1^ h^–1^), and H_2_ (0.3–4.4 μmol g^–1^ h^–1^) production rates than pristine CN. These studies attributed—usually by speculation—the superior photocatalytic activity of B-CN to a combination of enhanced CO_2_ adsorption, extended visible light absorption, better charge carrier separation, and easier access to surface redox sites. B-CN has also been investigated for photocatalytic NO_*x*_ removal (air purification) and presented increased NO/NO_*x*_ conversion percentage and selectivity to NO_3_^–^, compared to pristine CN.^24,32^ These studies suggested that enhanced photoactivity was due to extended visible light absorption (smaller bandgap), a more appropriate electronic band structure (specifically, a deeper conduction band), and longer-lived photoexcited electrons.

Despite the increased amount of research on B functionalization of CN, most studies incorporate B-CN as part of composites/heterojunctions, or co-doped with other metals and cocatalysts, or explore the effect of structure modification. In these studies, the testing conditions for photocatalytic CO_2_ and H_2_O reactions often involve the use of cocatalysts and hole scavengers. While this approach focuses on boosting catalytic efficiency, it makes it difficult to elucidate the sole effect of B functionalization on the properties of different CN materials. Arguably, many literature reports—although referring to the same material—are contradictory, especially with regard to chemistry and photoactivity. For instance, there is a lack of consensus about how B is integrated into the structure. B_C_ replacement is suggested by some studies,^28,29,33,34^ and B_N_ replacement by others.^20,35^ Some theoretical works also support grafting of B onto coordinated N atoms outside the heptazine rings.^36^ Interpretation of UV–visible spectroscopy results also varies, as B functionalization seems to have little effect,^27,29,34,37^ while other studies claim enhanced or extended light absorption.^28,31,33^ Finally, mainly due to different experimental setups and conditions used, a wide range of CO_2_ photoreducing activity is reported, and it is usually assigned to different properties (as discussed above).^24,26–31^

In this study, we aim to bridge this knowledge gap and investigate the effects of (i) B functionalization, (ii) B content, and (iii) choice of B dopant on the physicochemical, morphological, adsorptive, optoelectronic, and photocatalytic properties of bulk CN. For this purpose, we synthesize two sets of samples with varying B content using either amorphous boron or boric acid precursors, carry out advanced characterization (Fourier-transform infrared spectroscopy (FTIR), X-ray diffraction (XRD), X-ray photoelectron spectroscopy (XPS), electron paramagnetic resonance (EPR), solid-state nuclear magnetic resonance (NMR) spectroscopy, transmission energy microscopy (TEM), electron energy loss spectroscopy (EELS), N_2_ and CO_2_ adsorption–desorption, diffuse reflectance spectroscopy in the UV–visible (DRS UV–vis), steady-state photoluminescence (PL), ambient photoemission spectroscopy (APS), transient absorption spectroscopy (TAS)), theoretical calculations (using density functional theory (DFT)), and test our materials for the less extensively explored photocatalytic CO_2_ reduction and NO_*x*_ removal reactions. Some of the techniques used here have never been employed to characterize B-functionalized CN, namely, CO_2_ adsorption with related derivation of heat of adsorption, APS, and μs-TAS. These techniques are expected to provide additional and complementary structural information at scales ranging from atomic to bulk, and provide a much clearer picture of the materials. Our study demonstrates how B is integrated into the CN structure and isolates the effect of B functionalization on CO_2_ adsorption, light absorption, electronic band structure, and charge carrier behavior. In addition, we explain how these effects are linked to photocatalytic activity, and depend on the choice of B precursor and B content. Overall, our study clarifies several fundamental points of controversy in the literature around the nature and isolated effects of B functionalization, and provides the foundation for the rational development of improved photocatalysts.

## 2 Experimental Methods

The same single-step process was followed to synthesize pristine and B-functionalized CN samples, as described below. For the B-functionalized samples, two different precursors were used: amorphous boron and boric acid.

### 2.1 Synthesis of Pristine CN

Bulk CN was synthesized through the calcination of melamine. 10 g (0.08 mol) of melamine (C_3_H_6_N_6_, 99.0%, Sigma-Aldrich) was placed into an alumina crucible covered with a lid and heated in a muffle furnace up to 560 °C, with a heating rate of 10 °C min^–1^. The temperature was held for 4 h, and then the material was allowed to cool down naturally to room temperature. The obtained powders were ground using a mortar and pestle, and are denoted CN.

### 2.2 Synthesis of B-CN (B) Samples

B-functionalized CN samples were synthesized using melamine (C_3_H_6_N_6_, 99.0%, Sigma-Aldrich) and amorphous boron (B, >95.0%, Sigma-Aldrich) as precursors. 10 g (0.08 mol) of melamine and 0.02, 0.05, or 0.60 g (0.002, 0.005, or 0.06 mol) of boron were mixed using a mortar and pestle. The mixture was placed into an alumina crucible covered with a lid and heated in a muffle furnace up to 560 °C, with a heating rate of 10 °C min^–1^. The temperature was held for 4 h, and then the material was allowed to cool down naturally to room temperature. The obtained powders were ground using a mortar and pestle, and denoted B-CN (0.5B), B-CN (1B), and B-CN (3B) after using gradually higher amount of B as the precursor, respectively. The numbers in parentheses indicate the B content in at%, as determined from XPS analyses.

### 2.3 Synthesis of B-CN (BA) Samples

B-functionalized CN samples were synthesized using melamine (C_3_H_6_N_6_, 99.0%, Sigma-Aldrich) and boric acid (H_3_BO_3_, >99.5%, Sigma-Aldrich) as precursors, in the same process as described in Section 2.2. In this case, 0.17, 0.35, or 0.70 g (0.003, 0.006, or 0.01 mol, respectively) of boric acid were used as the B source. The obtained powders were ground using a mortar and pestle, and denoted B-CN (3BA), B-CN (5BA), and B-CN (11BA) after using gradually higher amount of boric acid, respectively. The numbers in parentheses indicate the B content in at%, as determined from XPS analyses.

### 2.4 Characterization Methods

We have employed FTIR spectroscopy, XPS, EPR, solid-state NMR, TEM in combination with EELS, powder XRD, and N_2_ sorption at 77 K to characterize the chemical and structural properties of the materials. To analyze the optoelectronic properties, we have used the following techniques: UV–vis spectroscopy, XPS, APS, steady-state PL, TAS, and EPR. Details on each technique can be found in the Supporting Information.

### 2.5 CO_2_ Adsorption

CO_2_ adsorption isotherms were measured immediately after the N_2_ adsorption–desorption measurements used for porosity analyses. The samples were degassed in situ at 393 K for 4 h down to 7 × 10^–5^ bar using the same 3Flex Porosity Analyzer described in the Supporting Information. CO_2_ gas (research grade, 99.999%, BOC) was used, and the adsorption isotherms were measured sequentially at 288, 298, and 308 K up to 1 bar. The isotherms were fitted using the dual-site Langmuir model^38^:

$$q_{j}^{*}=\frac{q_{\mathrm{sb},j}b_{j}p}{1+b_{j}p}+\frac{q_{\mathrm{sd},j}d_{j}p}{1+d_{j}p}$$ 1

$$b_{j}=b_{0,j}\exp\left(\frac{-\Delta U_{b,j}}{RT}\right)$$ 2

$$d_{j}=d_{0,j}\exp\left(\frac{-\Delta U_{d,j}}{RT}\right)$$ 3

where *q*_*j*_ * is the adsorbed amount of gas *j* at pressure *p* and temperature *T*, *b* and *d* are adsorption coefficients, *b*_0_, *d*_0_, Δ*U*_b_, and Δ*U*_d_ are constants, *R* is the universal gas constant, and *q*_sb_ and *q*_sd_ are saturation capacities. The Langmuir isotherm fitting was carried out with MATLAB R2022a (The Mathworks Inc.) using the in-house software package isothermFittingTool.^39^ Afterwards, the isosteric heat of adsorption (Δ*H*_ads_) was calculated by applying the Clausius–Clapeyron equation^40–42^ using the above dual-site Langmuir parameters, based on the equation^43^:

$$-\Delta H_{\mathrm{ads},j}=-\left[\frac{\frac{q_{\mathrm{sb},j}b_{j}\Delta U_{b,j}}{(1+b_{j}p{)}^{2}}+\frac{q_{\mathrm{sd},j}d_{j}\Delta U_{d,j}}{(1+d_{j}p{)}^{2}}}{\frac{q_{\mathrm{sb},j}b_{j}}{(1+b_{j}p{)}^{2}}+\frac{q_{\mathrm{sd},j}d_{j}}{(1+d_{j}p{)}^{2}}}\right]$$ 4

### 2.6 Photocatalytic CO_2_ Reduction

Liquid-phase CO_2_ photoreduction experiments were performed in a homemade setup (Figure S1) using a stainless steel photoreactor (212.1 cm^3^) equipped with a fused quartz window for irradiation. Approximately 30 mg of sample was added to 5 mL of H_2_O inside a 50 mL glass beaker, with a quartz-encapsulated magnetic stirrer. The beaker containing the suspension was placed inside the photoreactor under continuous stirring, and the system was placed under vacuum at approximately 0.02 bar to remove air. CO_2_ gas (40 cm^3^ min^–1^, research grade, 99.999%, BOC) was passed through the suspension via a quartz capillary tube for 40 min. Finally, the photoreactor was filled with approximately 1.7 bar of CO_2_ gas and sealed. The sample was then irradiated for 5 h using an LSH302 light source (LOT Quantum Design) equipped with a 300 W Xe arc lamp (UXL-302-O, Ushio) at a distance of 9 cm from the surface of the suspension (area of 8 cm^2^), providing a 160 mW cm^–2^ intensity at the surface. After the 5 h reaction, the gaseous products were detected and analyzed using a 7890B gas chromatographer (GC) (Agilent), equipped with a packed HayeSep Q column (Agilent J&W, 6 foot, 1/8 in., 2 mm, 80/100 SST) and a packed MolSieve 5A column (Agilent J&W, 6 foot, 1/8 in., 2 mm, 60/80, preconditioned) in series, a thermal conductivity detector (TCD), and a flame ionization detector (FID). The photocatalytic tests were repeated three times on fresh samples. Control experiments were performed: (a) without irradiation, (b) with no sample, and (c) under a N_2_ atmosphere.

### 2.7 Photocatalytic NO_*x*_ Removal

Photocatalytic NO_*x*_ removal tests were carried out in a purpose-made setup, built in accordance with the ISO (22197–1:2016) protocol. 50 mg of sample was dispersed in ethanol and sonicated. The dispersion was transferred to a 10 mL spray bottle and sprayed onto the surface of float glass (5 cm × 10 cm; 50 cm^2^) using an airbrush gun (Fengda Professional-130 with a 0.3 mm nozzle). The tests were performed under a continuous 1 L min^–1^ flow of air containing 3 ppm of NO, at 50% relative humidity and room temperature. The next five steps were followed sequentially while testing each sample: (i) 10 min under dark, (ii) 20 min under UVA irradiation with an intensity of 1.5 mW cm^–2^ using two 15 W lamps (FL15T8BLB, 352 nm, Sankyo), (iii) 10 min under dark, (iv) 20 min under visible (white) light with an intensity of 3.5 mW cm^–2^ using two 15 W lamps (F15W/T8/840, Sylvania), and (v) 10 min under dark. The average NO/NO_2_/NO_*x*_ levels observed over 1 min intervals were measured by using a Serinus 40 chemiluminescence NO_*x*_ analyzer (Ecotech). The average NO/NO_*x*_ removal (%), NO deposition velocity (*v*_d_, cm s^–1^), and NO quantum efficiency (QE, %) under both UVA and white light irradiation were calculated based on the equations:

$$\mathrm{NO}\;\mathrm{removal}(\%)=\frac{[\mathrm{NO}{]}_{\mathrm{initial}}-[\mathrm{NO}{]}_{\mathrm{final}}}{[\mathrm{NO}{]}_{\mathrm{initial}}}\times 100\%$$ 5

$${\mathrm{NO}}_{x}\;\mathrm{removal}(\%)=\frac{[{\mathrm{NO}}_{x}{]}_{\mathrm{initial}}-[{\mathrm{NO}}_{x}{]}_{\mathrm{final}}}{[{\mathrm{NO}}_{x}{]}_{\mathrm{initial}}}\times 100\%$$ 6

vd(cms−1)=log([NO]initial[NO]final)·FA 7

$$\mathrm{QE}(\%)=\frac{F\cdot ([\mathrm{NO}{]}_{\mathrm{initial}}-[\mathrm{NO}{]}_{\mathrm{final}})\cdot N_{\mathrm{AV}}}{V_{m}\cdot 1,000,000\cdot A\cdot p_{f}}\times 100\%$$ 8

where [NO]_initial_ and [NO_*x*_]_initial_ are the average levels (ppm) of NO and NO_*x*_ under dark, respectively, [NO]_final_ and [NO_*x*_]_final_ are the average levels (ppm) of NO and NO_*x*_ under 20 min of UVA or white light irradiation, respectively, *F* is the gas flow rate (cm^3^ s^–1^), *A* is the sample area (cm^2^), *N*_AV_ is Avogadro’s number (molecules mol^–1^), *V*_m_ is the molar volume at standard temperature and pressure (cm^3^ mol^–1^), and *p*_f_ is the photon flux on the sample surface under UVA or white light irradiation (photons s^–1^ cm^–2^).

## 3 Results & Discussion

### 3.1 Chemistry, Structure, and Morphology

For this study, along with a pristine CN reference sample, we synthesized two sets of B-functionalized CN samples using two B-containing precursors in different amounts: amorphous boron (B) (B-CN (B) samples) and boric acid (BA) (B-CN (BA) samples) (Figure 1). The numbers in the samples’ names correspond to the B content in at%, as determined from XPS analyses. The B-CN (BA) samples resemble pristine CN, albeit with a more intense yellow color. However, B-CN (B) samples become darker as the amount of the B precursor increases. This color could be linked to the dark brown color of the amorphous B precursor and could be a sign of (unreacted) B present in the B-CN structure.

**Figure 1:**
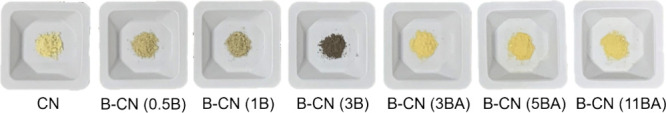
Photographs of the as-prepared and B-functionalized CN samples.

To check whether the core CN structure remains unchanged with B functionalization, we performed FTIR, XRD, and N_2_ adsorption–desorption (77 K) measurements on the materials (Figure 2). In the FTIR spectra (Figure 2a), the typical N–H/O-H (3000–3300 cm^–1^), C=N (1500–1700 cm^–1^), C–N (1250–1450 cm^–1^), and triazine ring (750–900 cm^–1^) bands of CN are present for all materials, as expected.^4,31,32,44^ These observations point to the successful synthesis of CN-based materials. XRD analyses (Figure 2b) confirm that all materials retain the stable tri-s-triazine structure of CN. In addition, they all exhibit the main and unshifted (100) and (002) planes, and secondary (101), (300), and (004) planes of CN. We conclude that B functionalization does not affect the planar *d*-spacing or π-stacking of CN. However, some B-CN samples show a decrease in (002) peak intensity (e.g., B-CN (11BA), the lowest), which may indicate a more defective atomic structure. To examine the porosity of the materials, we performed N_2_ adsorption–desorption measurements at 77 K (Figures 2c and S2a). All materials show Type II/III isotherms with Type H3/H4 hysteresis loops, typical of materials with narrow, slit-like pores.^45^ The textural properties, including BET area (*S*_BET_), total pore volume (*V*_tot_) and micropore volume (*V*_micro_), are derived from the N_2_ adsorption (77 K) isotherms (Figure 2c and Table 1). All samples have similar *S*_BET_, except B-CN (11BA)—the sample with the highest degree of functionalization—which shows the highest BET area and pore volume. These values are in accordance with literature for bulk CN, and others have also reported negligible change in *S*_BET_ with B functionalization in bulk form. The B-CN (11BA) sample is among the most porous B-functionalized bulk CN materials, surpassing some nanotubes.^7,26,28,29,31,32^ Considering *V*_micro_ and the ratio *V*_micro_/*V*_tot_, all samples are predominantly mesoporous, with B-CN (11BA) exhibiting the largest proportion of micropores. In summary, the introduction of B and the choice of B precursor on their own do not affect the porosity of the materials. Instead, it is the amount of B functionalization (i.e., 11 at. %) that has a significant effect, enhancing the surface area and microporosity.

**Table 1:** Textural Properties and Isosteric Heat of Adsorption of Pristine and B-Functionalized CN Samples^a^

sample	*S*_BET_ (m^2^ g^–1^)	*V*_tot_ (cm^3^ g^–1^)	*V*_micro_ (cm^3^ g^–1^)	*V*_micro_/*V*_tot_ (%)	isosteric Δ*H*_ads_ (kJ mol^–1^)
CN	17	0.076	0.012	15.8	16.7
B-CN (0.5B)	13	0.066	0.010	15.2	16.1
B-CN (1B)	22	0.076	0.015	19.7	12.4
B-CN (3B)	10	0.046	0.007	15.2	11.4
B-CN (3BA)	13	0.053	0.009	17.0	12.8
B-CM (5BA)	14	0.045	0.009	20.0	11.7
B-CN (11BA)	40	0.106	0.033	31.1	17.8

a The textural properties were derived from N_2_ adsorption measurements at 77 K, including the BET area (*S*_BET_), total pore volume (*V*_tot_), micropore volume (*V*_micro_), and micropore/total pore volume ratio (*V*_micro_/*V*_tot_). The heat of adsorption (Δ*H*_ads_) was calculated using the Virial fit on CO_2_ adsorption data at three temperatures (288, 298, and 308 K) (Figures S9 and S10).

**Figure 2:**
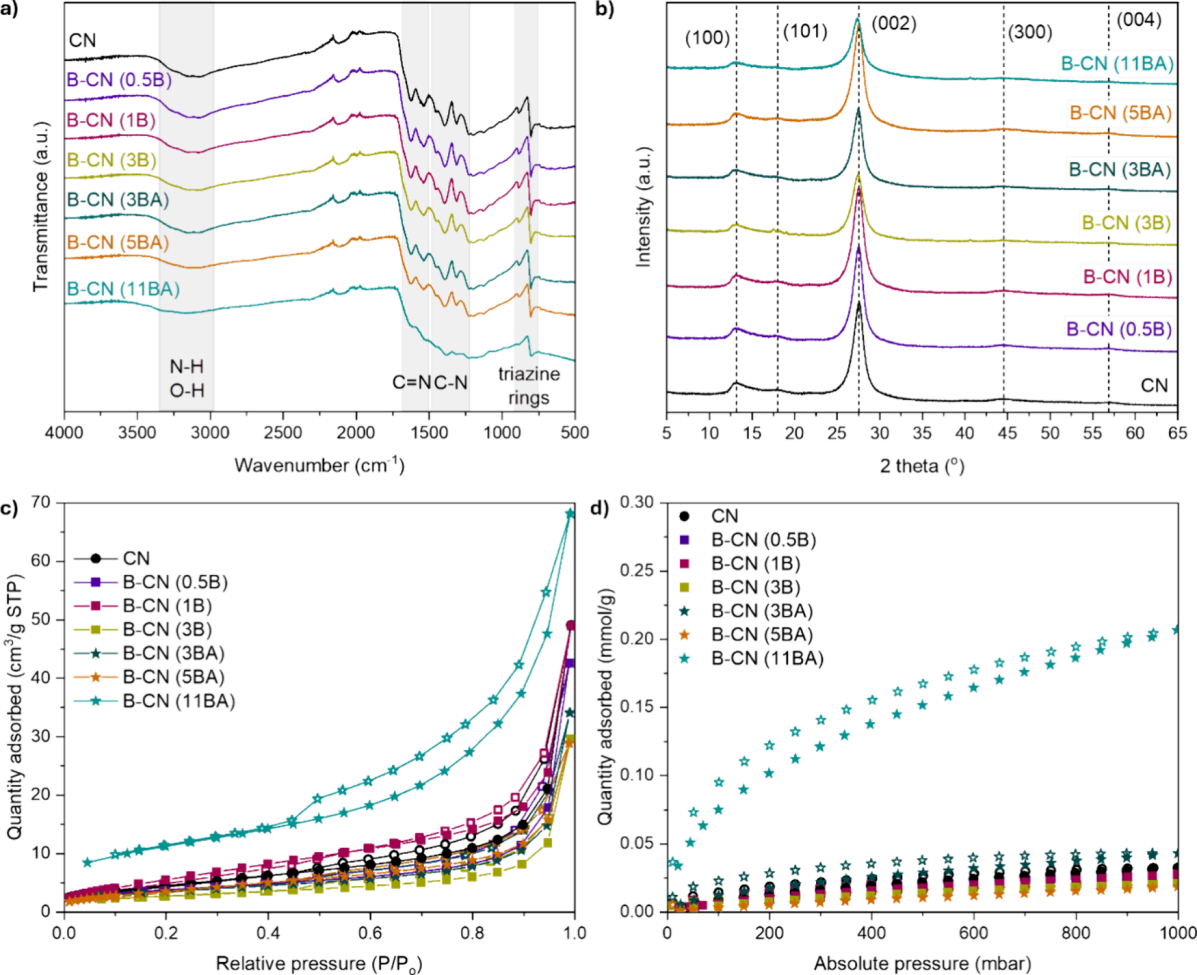
(a) FTIR spectra with highlighted IR bands, (b) powder XRD patterns, (c) N_2_ adsorption–desorption isotherms (77 K), and (d) CO_2_ adsorption–desorption isotherms (298 K) of pristine and B-functionalized CN samples. Filled symbols represent adsorption, and empty symbols represent desorption in parts (c, d). For clarity, the N_2_ and CO_2_ adsorption–desorption isotherms are also plotted on a logarithmic scale in Figure S2.

To explore the chemistry of the B-CN materials and identify if B is incorporated into the structure, we performed XPS, NMR, and EPR analyses. Initially, to confirm the successful B functionalization of our materials and investigate the chemical environment around B atoms, we carried out XPS measurements, focusing on the B 1*s*, C 1*s*, N 1*s*, and O 1*s* core-level spectra. Through XPS elemental composition analysis (Figure 3a), we verify the presence and actual composition of B in our B-CN (B) and B-CN (BA) samples: 0.5–3 at. % for B-CN (B) samples and 3–11 at. % for B-CN (BA) samples. The nominal B content for both precursors was 3, 5, and 10 at. %. By comparing the nominal (i.e., expected) versus the actual B concentrations from XPS, it appears easier to functionalize and regulate B content using BA. This situation is likely due to the chemical affinity between the acid and the basic melamine. Using a higher amount of B as the precursor does not result in a linearly higher amount of B content in the final sample. For B-CN (B) samples, the maximum B content is 3 at%. Overall, the concentration of O atoms seems to follow that of B atoms, up to 5 at%, regardless of the B precursor. This observation could potentially indicate that B is bonded to O upon incorporation—although the disproportional B and O contents in B-CN (11BA) do not necessarily support this theory. We note that, as seen in Figure 3a, O (4 at%) is present as a defect even in pristine CN. Hence, it is possible that B atoms bond to O atoms already present in the CN structure, as well as to ‘new’ ones introduced in the synthesis. From 0.5 to 5 at% of B (and O), we see an analogous decrease in both C and N content. For 11 at% B, the larger decrease in the N content than in the C content could indicate that a high amount of B functionalization comes mostly at the expense of N atoms. The difference between C and N contents remains small though and is within the error of ±2% on these measurements. The B 1*s* XPS spectra (Figures 3b and S3) show that: (i) the main chemical bonding of B atoms is B–O, and (ii) the choice of B precursor affects the chemistry. In general, all samples—except B-CN (11BA)—exhibit a main peak at the same binding energy (∼192.5 eV) that can be deconvoluted into two peaks (∼193 and ∼192 eV), due to the asymmetry of the peak and the full width at half-maximum (FWHM) values (Table S1). We assign the deconvoluted peak at ∼192 eV to B–O, while the secondary deconvoluted peak at ∼193 eV could be potentially assigned to B–O–H bonding after comparing to the boric acid spectrum. Both B chemical environments are equally formed in the B-CN (5BA) sample, while the ratio between the two fluctuates for the other samples. In the case of B-CN (11BA), it seems that there is only a single peak at ∼192 eV and hence a single B–O coordination. On the other hand, B-CN (B) samples exhibit, other than the main B–O peak (∼192.5 eV), a secondary peak at ∼187.5 eV, assigned to metallic B^0^. The intensity of this secondary peak increases with higher B content (3 at. %) but is absent for the B-CN (3BA) sample, which has the same B content. Therefore, we are confident that the secondary peak is not an artifact from B reduction due to high-energy X-ray exposure. Rather, it is caused by the agglomeration of (unreacted) B atoms in B-CN (B) samples. In the C 1*s* and N 1*s* XPS spectra (Figure S4a,b), all materials exhibit the typical C–N=C bonding, along with adventitious C–C, bridging N-(C)_3_ groups, C–O/C=O, and −NH/NH_2_ moieties. We do not observe any shifts in binding energies, nor any change in the deconvoluted peaks. Hence, B functionalization does not affect the chemical environments around C and N atoms; however, it does seem to impact the O chemical environment. The single O–C (∼532.5 eV) bonding in pristine CN changes to deconvoluted O–B (532–533 eV) and O=C (531–531.5 eV) bonds in B-CN samples, regardless of the B precursor (Figure S4c). Combining our XPS elemental composition analysis with the XPS core-level spectra, we suggest the following aspects in terms of the chemical environment in the B-CN materials. First, B is linked to O–and also exists in B^0^ clusters in B-CN (B) samples. Second, some O atoms pre-exist in the CN structure by replacing N atoms (O_N_) and creating reactive −OH groups. Third, B atoms do not replace N atoms or C atoms (no B_C_ or B_N_) and do not form B–N or B–C bonds. These findings are only partly in accordance with the literature. The same B 1*s* peak at ∼192 eV has often been reported in the literature, but it was almost always assigned to B–N bonding (B_C_ substitution), and occasionally to B–C bonding (B_N_ substitution).^4,8,13,18,24,25,28,29,44,46^ In all cases in the B-CN literature, this assignment is based on the same assumption that the B 1*s* peak in B-CN is more electronegative than the B–N core (190 eV) in hBN^47^ and less electronegative than the B–(N)_3_ bond (193 eV) in Kawaguchi’s B-CN.^48^ This literature assignment is contradictory to the fact that B 1*s* peaks in the range 191.5–193 eV are typically assigned to B–O environments.^49–52^ Therefore, our observation that B is linked to O (and can also exist in B clusters) and B atoms do not replace N atoms nor C atoms and do not form B–N or B–C bonds, differs from previous literature. For our B-CN (B) materials, we are in agreement with a study where CN was decorated with elemental boron and suggested the formation of B clusters.^25^ Although few, some previous reports containing O 1*s* spectra suggest the creation of C–O and O–H bonds. These reports thus support our claim that O pre-exists in the CN structure, replacing N atom sites.^11,26,31,53^

**Figure 3:**
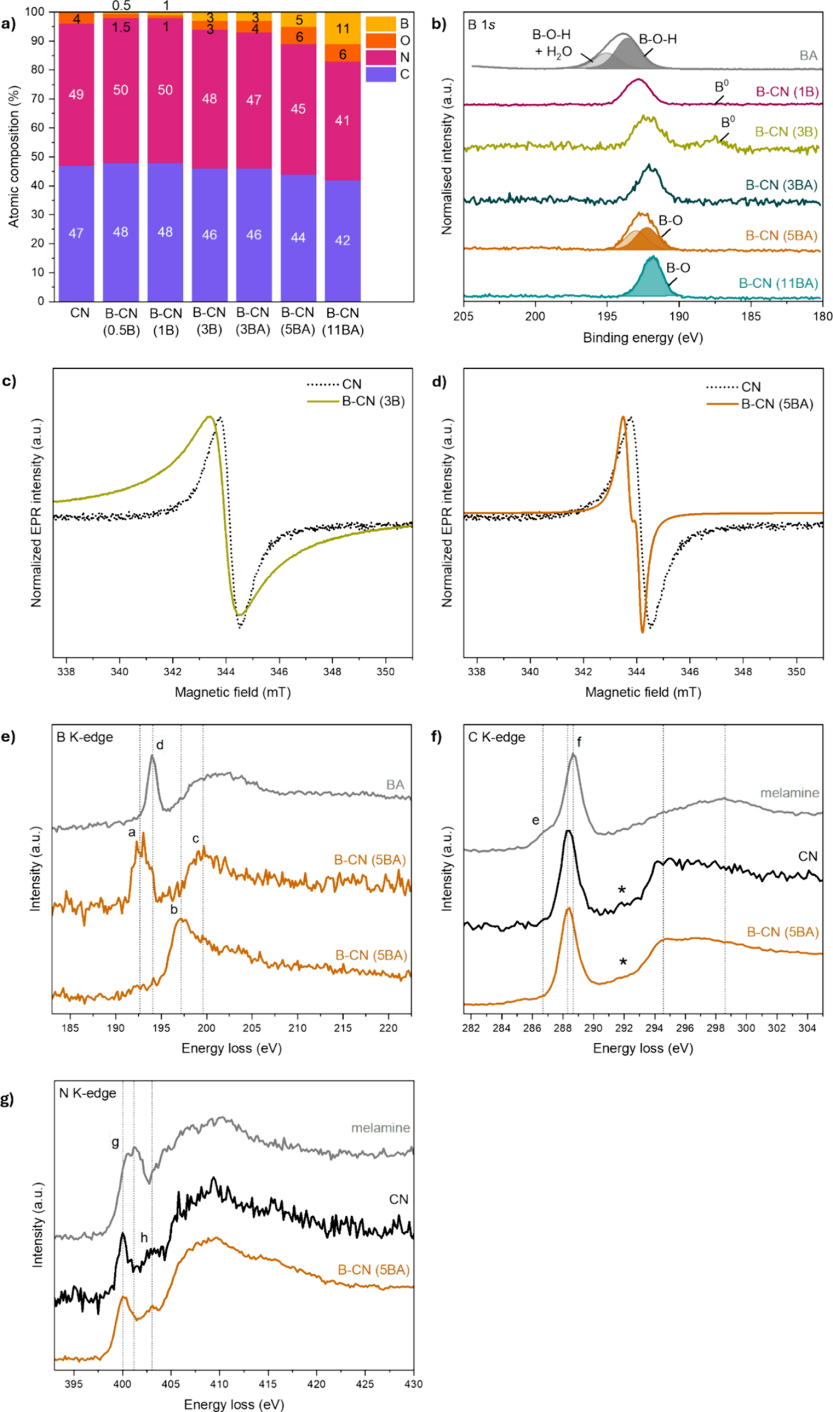
(a) Elemental composition of pristine CN and B-CN samples, as derived from XPS analyses, (b) XPS B 1*s* spectra of B-CN (1B), B-CN (3B), B-CN (3BA), B-CN (5BA), and B-CN (11BA), (c, d) normalized EPR measurements at room temperature in air for B-CN (3B) and B-CN (5BA), respectively, compared to normalized EPR data of CN taken under the same conditions, (e) B K-edge EEL spectra taken from boric acid and from different areas of B-CN (5BA) sample, (f) C K-edge EEL spectra of melamine, CN, and B-CN (5BA), and (g) N K-edge EEL spectra of melamine, CN, and B-CN (5BA).

To provide another level of confidence to our claims, we performed EPR measurements at room temperature in air. The normalized and non-normalized EPR spectra of all materials, as obtained at room temperature in air, are presented in Figure S5. The EPR signal in almost all B-CN samples is similar to that of the pristine CN (Figure S5a–e). According to the literature,^54^ the EPR signal of CN materials originates from unpaired electrons in the localized π-conjugated structure. In contrast, samples B-CN (3B) and B-CN (5BA) exhibit differences in both the intensity and the shape of the EPR signal. B-CN (3B) shows a broader EPR signal (Figures 3c and S5f), which could be explained by the agglomeration of B atoms in the structure, in accordance with our XPS results. The reason the rest of the B-CN (B) samples do not show this difference in signal, despite exhibiting the same chemistry, would lie in the concentration of such species: B-CN (3B) has the strongest XPS signal for B^0^, and therefore the highest concentration. B-CN (5BA) is the only sample among B-CN (BA) samples to show the formation of a radical in the EPR signal (Figures 3d and S5g). The creation of a second deconvoluted peak (potentially secondary B–OH bonding) at 193 eV in the B 1*s* spectrum of B-CN (5BA) (Figure 3b) could be related to the cause of radical formation in the EPR signal. The EPR signal intensities of the B-CN (3B) and B-CN (5BA) samples are also higher than the other samples (B-CN (3B): ×3, B-CN (5BA): ×200) (Figure S5h,i). This could indicate the existence of more defects, hence increasing the concentration of unpaired electrons in the π-conjugated structure of the heptazine rings (which are the source of the EPR signal).

To support our suggestions on the chemistry of the B-functionalized materials and to provide yet another level of confidence in the conclusions, we carried out solid-state NMR measurements. ^11^B MAS NMR spectra of the B-CN samples are presented (Figure 4) along with spectra for the boron precursors, boric acid (Figure 4a) and boron (Figure 4b). Spectra were also recorded using a spin echo pulse sequence to ensure that no very broad lineshapes^50^ were lost in the conventional MAS NMR spectra, and these showed no significant differences. For the B-CN (BA) samples, two sets of signals are seen, corresponding to trigonal (12–19 ppm) and tetrahedral (2 to −4 ppm) boron environments. The relative integrated signal intensities for the two different coordination environments of B show similar levels of tetrahedral B for the two samples with lower BA content. However, the level of tetrahedral B increases at the highest level of B doping (Table 2). The presence of significant amounts of tetrahedral B indicates that different types of B species are present in addition to any substitution into the CN layers, as has been suggested in previous works.^11,26^ The signal attributed to trigonal B exhibits a characteristic second-order quadrupolar broadened lineshape,^50^ suggesting a much larger quadrupolar coupling constant (*C*_Q_), as would be expected from the lower site symmetry. The signal in this region is similar (although not identical, as discussed below) to that seen for boric acid itself. This observation suggests a similar local structure and the likely presence of B–O (rather than B–N) bonds. In contrast, the signals in the tetrahedral region appear much sharper, as expected, given the higher local site symmetry, although several overlapping resonances are present.

**Figure 4:**
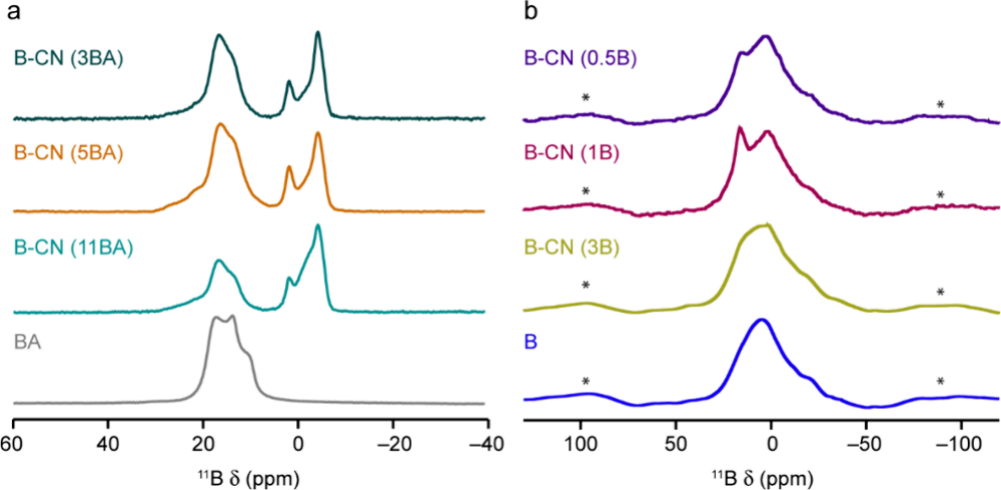
^11^B (14.1 T, 14 kHz) MAS NMR spectra of (a) B-CN (BA) samples and boric acid, and (b) B-CN (B) samples and boron, acquired using a short flip angle. Spinning sidebands are marked with *.

**Table 2:** Relative Integrated Signal Intensities (Expressed as %) for the Trigonal and Tetrahedral B Species in the ^11^B MAS NMR Spectra (Acquired with a Short Flip Angle) of the B-CN (BA) Samples (Figure 4)­^a^

sample	trigonal B (%)	tetrahedral B (%)
B-CN (3BA)	61 (1)	39 (1)
B-CN (5BA)	65 (1)	35 (1)
B-CN (11BA)	46 (1)	54 (1)

a The values in parentheses are the estimated uncertainties.

To remove the second-order quadrupolar broadening, ^11^B MQMAS NMR spectra were acquired (Figure 5). These spectra confirm the presence of a single type of trigonal B species (although this is broadened by a longer-range disorder). The signals from tetrahedral B display very little quadrupolar broadening, but at least three overlapped signals are present (confirming that a range of different B environments exist in these materials). NMR parameters are extracted from the signals seen for B-CN (11BA) and presented alongside those of BN from the literature^55^ (Table 3). It is clear that the trigonal B species present in the B-CN (BA) samples is similar to boric acid itself but has slightly different NMR parameters, including a smaller quadrupolar interaction. However, when compared to the NMR parameters for BN in the literature, this trigonal B species has a much lower δ_iso_ (and so appears at a much lower δ_1_ value in the ^11^B MQMAS NMR spectrum). This is also in good agreement with previous work^50^ investigating the formation of porous BN, where BN_3_ and BO_3_ species were seen at δ_1_ = 72 and 45 ppm (14.1 T), respectively. Hence, the trigonal B species present is more likely to be BO_3_ (or perhaps BO_2_N), rather than the BN_3_ environment expected if all B were substituted directly for C in the CN layers.

**Figure 5:**
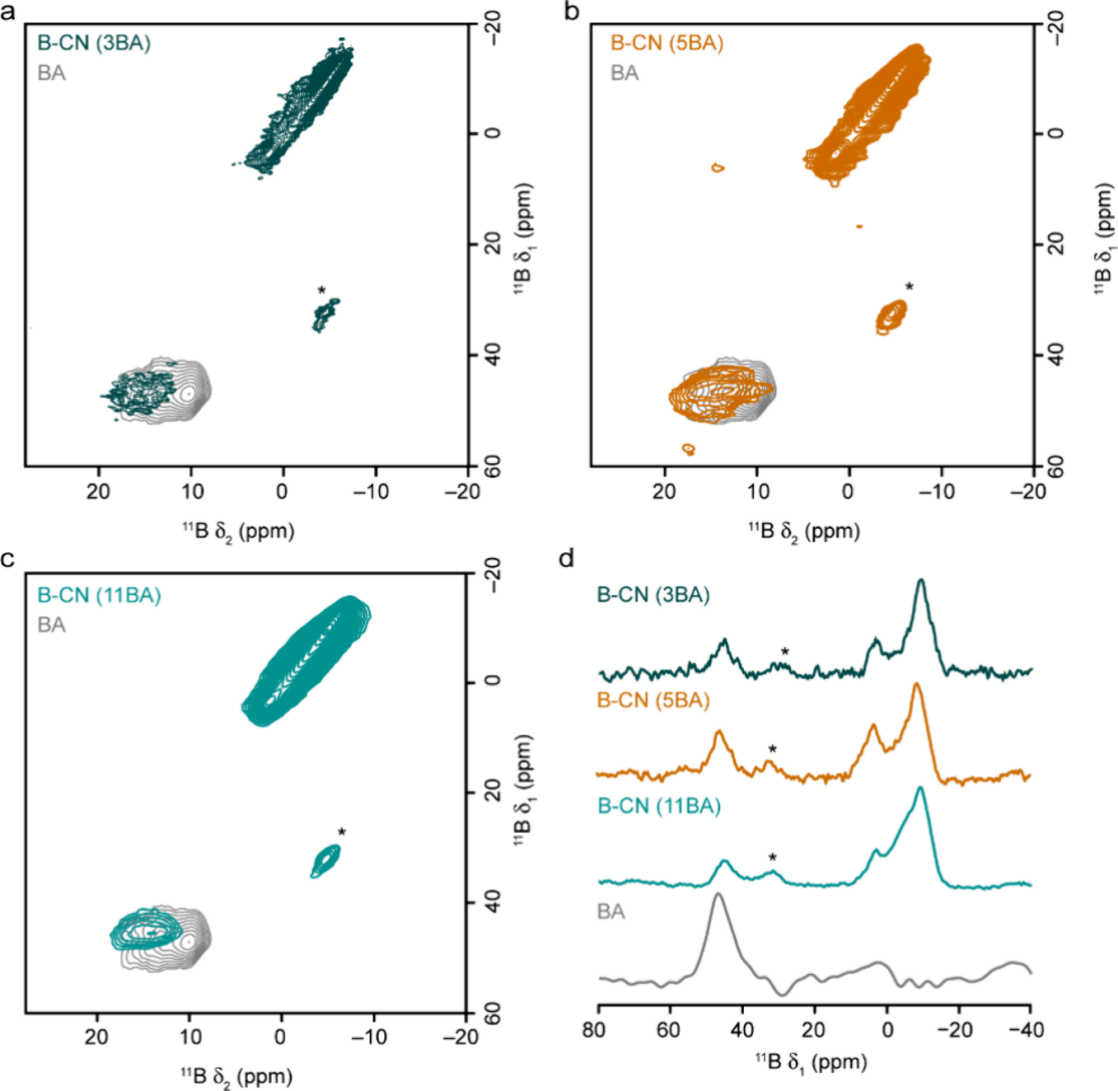
^11^B (14.1 T, 14 kHz) MQMAS NMR spectra of B-CN (BA) samples for (a) B-CN (3BA), (b) B-CN (5BA), and (c) B-CN (11BA), each overlaid with the corresponding spectrum for boric acid (BA, shown in gray), with (d) corresponding isotropic projections. Spinning sidebands are marked with *.

**Table 3:** NMR Parameters Extracted from the ^11^B (14.1 T) MQMAS NMR Spectra of B-CN (11BA) and Boric Acid (Figure 5c), along with Those from the Literature for Hexagonal BN^a^

	δ_1_ (ppm)	δ_2_ (ppm)	δ_iso_ (ppm)	*P*_Q_ (MHz)
B-CN (11BA)	–9.2	–4.6	–4.4 (5)	0.5 (2)
	–5.9	–3.1	–2.9 (5)	0.5 (2)
	3.2	1.4	1.5 (5)	0.3 (2)
	45.0	14.9	18.8 (10)	2.4 (3)
boric acid	47.0	10.4	17.8 (10)	3.3 (3)
hBN	71.6^b^	24.7^b^	30.4	2.9

a Values in parentheses show the estimated uncertainties.

b These values are predicted from the NMR parameters in the literature for hexagonal BN.^55^

The ^11^B MAS NMR spectra of the B-CN (B) samples are shown in Figure 4b. The spectra appear very different to those of the B-CN (BA) samples, containing a broad lineshape that spans the regions where signals for both trigonal and tetrahedral B are seen (Figure 4a). The spectra shown in Figure 4b were acquired with a short flip angle to ensure that the results are quantitative. However, only small changes in the spectral lineshapes were seen when spectra were acquired using a spin echo, and with varying recycle intervals, indicating that there are no further “invisible” signals for these samples. All spectra are similar (though not identical) to those seen for elemental B (Figure 4b). This feature suggests that the B environment in this set of B-CN samples is similar to that in elemental B and is very different from that in the samples prepared with BA. To understand the origin of the spectral broadening (i.e., whether it results from a quadrupolar interaction or from a distribution of local environments), ^11^B MQMAS spectra were acquired for B-CN (3B) and boron (Figure 6). In both cases, similar spectral lineshapes are seen, confirming the similar nature of the B environments in the two samples. The signals (overlapped even in this 2D spectrum) do not lie along an axis parallel to δ_2_, which would suggest the presence of quadrupolar broadening. Instead, they align along an axis that confirms that a distribution of shifts is present, and the intense sidebands also confirm the presence of significant shielding anisotropy. The ^11^B MAS NMR spectrum seen for boron agrees with that in previous literature.^56^ In this earlier work, the authors suggested that the broader lineshapes seen result from the defects in the structure (i.e., missing B atoms that not only formally lift any equivalencies leading to disorder, but also give rise to changes in bulk magnetic susceptibility). It is clear, however, that the similarity of the B-CN (B) spectra to that of boron suggests limited substitution of B for C in the CN layers, but rather the presence of B-like clusters within the material.

**Figure 6:**
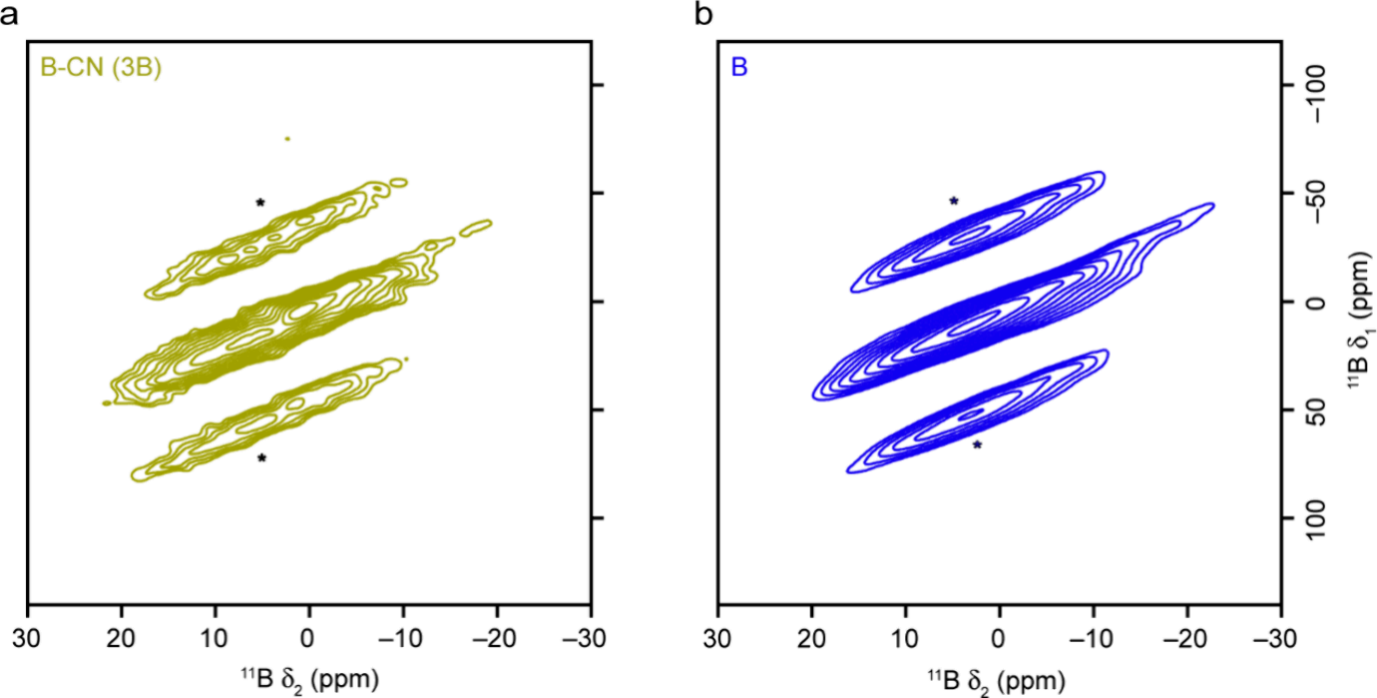
^11^B (14.1 T, 14 kHz) MQMAS NMR spectra of (a) B-CN (3B) and (b) boron. Spinning sidebands are marked with *.

We measured the ^13^C MAS and CP MAS spectra of the B-CN (B) and B-CN (BA) samples, along with the corresponding spectra for CN (Figure 7). The MAS spectra (recorded with a spin echo to remove the probe background signal) have fairly poor sensitivity, largely as a result of slow *T*_1_ relaxation and consequently long recycle intervals that have to be used. Both these spectra and those acquired using CP from ^1^H contain two distinct signals (at 165 and 157 ppm). No significant differences are seen in the relative intensities of the two signals with increased B content (at least within the level of the noise in the spin echo spectra). There are also no differences between samples made using BA and B (perhaps with the exception of B-CN (11BA)), despite the very different ^11^B spectra seen for the two sets of samples. When the similarity of these spectra to those of CN is also considered, there is little evidence for any substitution of B into the CN layers for either set of samples. This conclusion contradicts previous works where similar spectra were seen.^11,26^ For the unsubstituted CN, the integrated intensities of the two signals have different behavior as a function of the CP contact time (Figure S6). Indeed, the signal at 165 ppm builds up more quickly, suggesting closer proximity to ^1^H (on average). The ^1^H MAS NMR spectra are similar for all B-CN samples, irrespective of how they are prepared. The spectra are dominated by a signal at 8.6 ppm, attributed previously to the NH_2_ groups on the layer edges (and at defect sites)^11^ (Figure S7). An additional signal is seen at 3.6 ppm (present at differing levels in each sample, including that of CN). This signal has been attributed in previous works to water (or possibly OH groups on the layer edges)^11^ and varies in both intensity and width with B substitution. However, the low resolution of ^1^H spectra at these slower MAS rates prevents a more detailed spectral analysis, and perhaps the most important conclusion is that no significant new environments are apparent with B functionalization. The disordered and defective nature of CN, even prior to any B integration, makes the unambiguous assignment of NMR signals challenging. Hence, there seems to be confusion over the interpretation of ^13^C and ^11^B NMR spectra of B-CN-type materials in previous literature.^11,13,26^ For CN, the two ^13^C signals seen have been previously assigned as those linked to amino groups (i.e., CN_2_(NH_*x*_), where *x* = 1 or 2) at 165 ppm, and those within the CN layers (i.e., CN_3_) at 157 ppm.^13,26^ Such an assignment is consistent with the CP buildup behavior, which suggested that the signal at higher shift was closer to ^1^H. Literature results for ^13^C MAS NMR spectroscopy of melamine show signals at 167.5 and 169.2 ppm, resulting from CN_2_(NH_2_) groups. Similar studies of melem show signals at 164.3 and 166.4 for similar species, in addition to two signals at 155.1 and 156.0 from CN_3_ groups.^57^ However, for the spectrum of CN, the ratio of the signals at 165 and 157 ppm is ∼53:∼47. This ratio suggests that a large number of CN_2_(NH_2_) groups would have to be present—many more than would be possible if any extended layer structure was present (Figure 7a).

**Figure 7:**
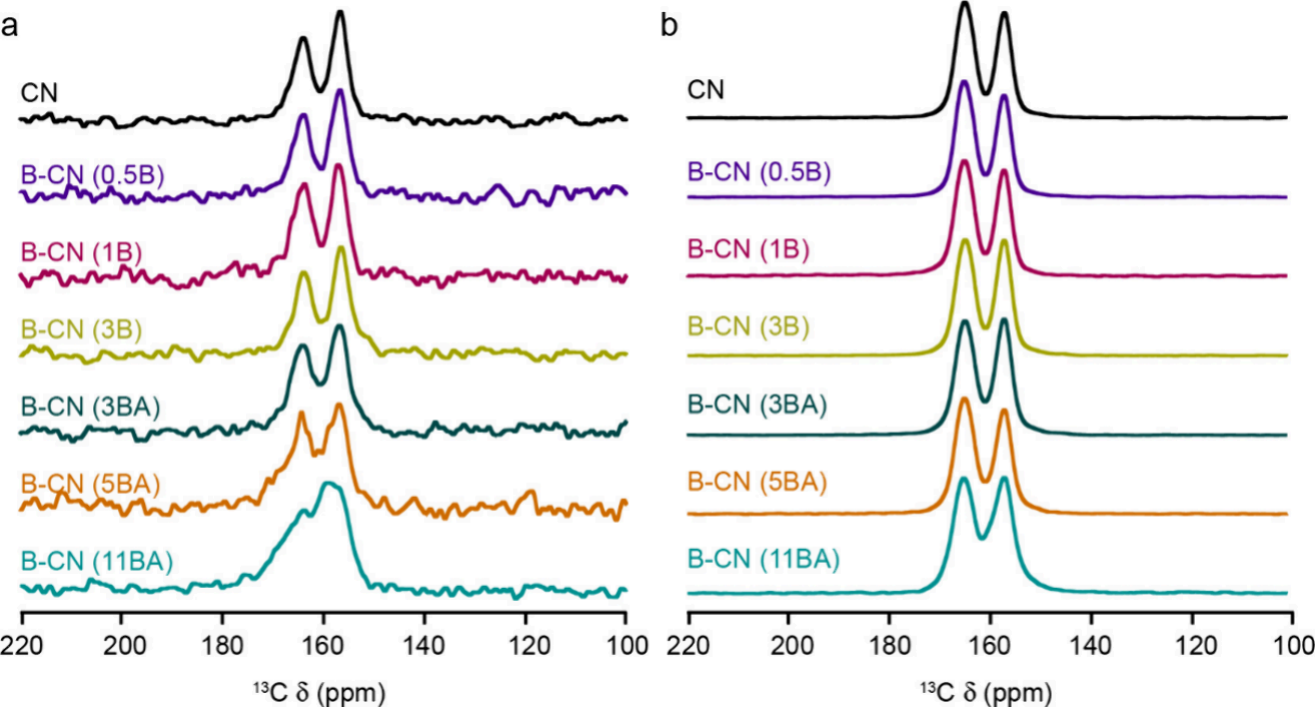
^13^C (9.4 T, 14 kHz) MAS NMR spectra of pristine and B-functionalized CN samples acquired using (a) spin echo and (b) CP from ^1^H.

To gain further insight, we carried out DFT calculations for two models of CN: the first being an idealized structure^57^ (Figure S8a) and the second a more disordered material^58^ (Figure S8b). We note that in both cases, the layers are fully connected (i.e., no NH_2_ groups are present). The predicted ^13^C chemical shifts are given alongside those predicted for the known crystal structure of melem^55^ in Table S2. C*N*_3_(*i*) is used to distinguish C that are bound only to N within one melem-like subunit (internal), and C*N*_3_(*e*) for those C that are bound to one N in a different subunit (external). The DFT-predicted shifts for the six distinct C species in melem agree well with the four signals seen in the ^13^C MAS NMR spectrum,^55^ predicting overlap of signals resulting from C1/C2 and C5/C6, and confirming the preliminary signal assignments in earlier work. The shifts calculated for CN_2_(NH_2_) groups are similar to the signal at 165 ppm in the experimental spectrum of CN. However, the calculated shifts for the two models of CN also predict two sets of signals, with different shifts for C*N*_3_(*e*) and C*N*_3_(*i*). While the absolute predicted shifts do not exactly match experiment (which is unsurprising given the idealized nature of the models used, in particular, the ordered model in Figure S8a), it seems most likely that the signal at 165 ppm in the experimental spectra results from the overlap of signals from both C*N*_3_(*e*) and CN_2_(NH_2_) groups (in fact from all C*N*_2_(*e*) sites, whether connected to a second melem-like subunit or an NH_2_ group). This also accounts for the different CP buildup behavior seen in Figure S6 and is consistent with an extended layered structure (and the decreased number of CH_2_(NH_2_) signals this would give).

Additional DFT calculations were carried out to predict the NMR parameters that might be observed if B were substituted within the CN layers. Six models were constructed, with B substituting for each of the four distinct C sites in the ordered model of CN (Figure S8a), and protonation of one of the neighboring NC_2_ (i.e., not any neighboring NC_3_) atoms. Although this model is less realistic than that for the disordered structure, it was chosen for these preliminary calculations owing to the smaller cell size. The relative energies of the six models are compared (Table S3). Substitution onto the C*N*_3_(*e*) sites appears more favorable than onto the C*N*_3_(*i*) sites, although substitution onto C*N*_2_(*e*) sites bonded to NH_2_ (i.e., CN_2_(NH_2_) sites) is not tested by these idealized models. There is a significant structural change to the CN layers in this ordered model when B is substituted on any site (Figure 8) for a model where B substitutes for C4 and the neighboring N5 is protonated. The B substitution results in a significant distortion of the melem-like rings, with distinct puckering and bending observed. Hence, the structure is more like that of the disordered model of CN, as shown in Figure S8b. Table S3 also shows the calculated ^11^B NMR parameters for the six models of substituted ordered CN. These are similar for substitution onto each of the types of C site, with δ_iso_ values between 24 and 29 ppm and moderate *C*_Q_ values of ∼3 MHz (as expected for trigonal B). We show the effect on the ^13^C δ_iso_ values for ordered CN (shown in red) of B substitution (shown in blue), as well as predicted values for disordered CN (in green) (Figure S9). On average, a decrease in shift is seen upon B substitution, suggesting that peaks in B-CN materials would be broadened and moved towards lower shift. We note that these calculations were carried out using an idealized model of CN and are, as such, indicative only. Further work, involving larger and more disordered models containing CN_2_NH_2_ defects, would be required to obtain more than the qualitative insight afforded here. However, the present results are consistent with the suggestion that B substitution into the layers would result in more significant changes in the ^13^C NMR spectra than are seen experimentally (Figure 7).

**Figure 8:**
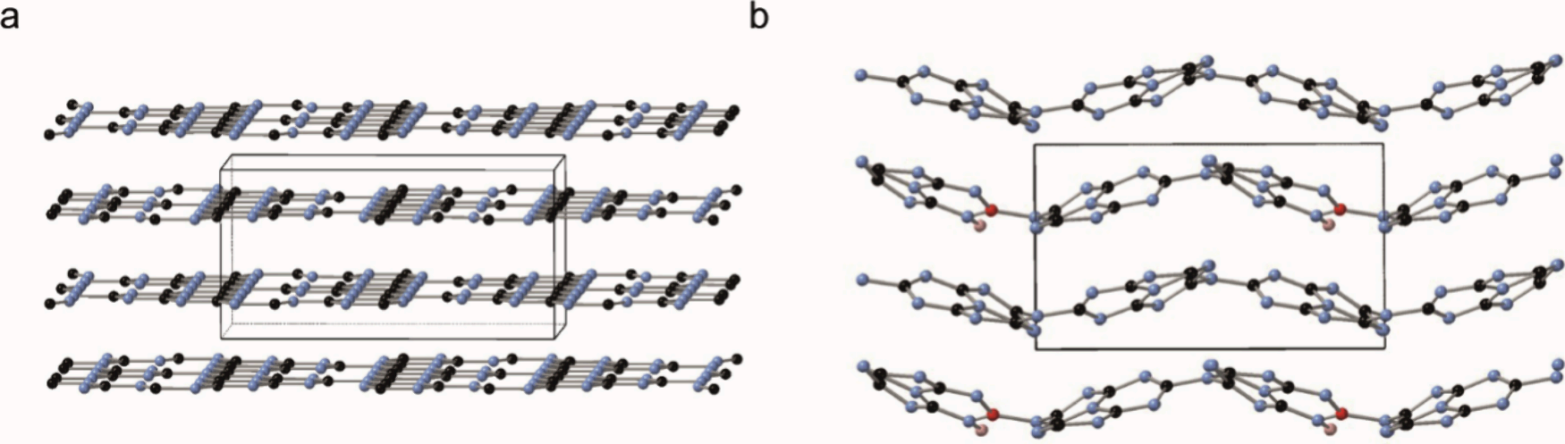
Optimized (using DFT) structural models for (a) ordered CN and (b) ordered CN with B substituted on one C4 site and protonation of the neighboring N5. (carbon = black, nitrogen = blue, boron = red, and hydrogen = pink).

By combining our XPS, EPR, NMR analyses, and DFT calculations, we suggest possible chemical structures for the B-CN (B) samples (Figure 9a) and B-CN (BA) samples (Figure 9b). In general, we suggest three main ‘events’. First, −OH groups are formed at the edges of the CN layers, attached to C atoms. Second, N atoms are protonated, forming amine groups (=NH and −NH_2_) in basal planes and at the edges, causing a discontinuation of the CN structure. Third, B atoms are integrated via B–O bonds through O_N_ substitution in basal planes. In addition to these, in the B-CN (B) samples, B clusters are present within the basal planes, causing further discontinuation and defects in the CN structure. In the B-CN (BA) samples, a secondary B–O environment exists within the basal planes, potentially through B–OH bonds, causing further changes to the CN structure.

**Figure 9:**
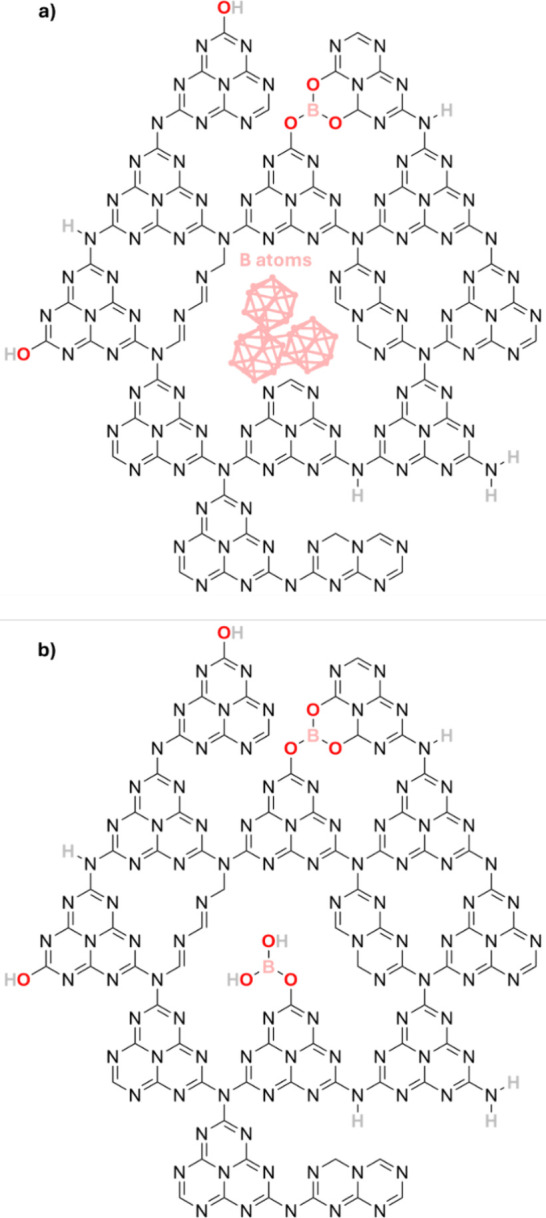
Schematic representations of proposed chemical structures for (a) B-CN­(B) and (b) B-CN­(BA), as suggested by XPS, EPR and NMR analyses, and DFT calculations. Included in the structures is the presence of N–H, C–/=O, and V_N_ defects, which are inherent to pristine CN structure, as well as B–O bonding and agglomerated B atoms resulting from B functionalization.

We used TEM to examine the morphology of the pristine and B-functionalized CN materials (Figure 10a,b). All materials exhibit a wide range of morphologies including particle clusters, amorphous phases, nanosheets, and nanotubes. The samples consist mostly of amorphous clusters, although the B-CN materials seem to consist of smaller and more dispersed clusters compared to pristine CN. Using EELS, we investigated the homogeneity of B functionalization at the nm-μm scale, and the coordination environment around the B, C and N atoms. We selected the sample B-CN (5BA) for EELS K-edge measurements as, based on our previous analyses, it shows a diverse B chemical bonding and has a detectable B content (5 at%). During EELS measurements, by scanning different pixels/areas in the same sample, we can see there is inhomogeneity in B functionalization, as B is not found in every particle/particle cluster. Furthermore, in the pixels/areas where B does exist, it exhibits different coordination environments—in accordance with the models proposed in Figure 9. These different chemical environments result in several peaks in the B K-edge spectrum of B-CN (5BA). The sharp peak at 192.8 eV (a) is assigned to the B 1*s* → π* transitions, and broader peaks at 197.0 eV (b) and 199.0 eV (c) to the B 1*s* → σ* transitions (Figure 3e). We note here that the spectra differ between those of B-CN (5BA) and boric acid (reference sample). In the B K-edge spectrum of boric acid, the main B 1*s* → π* peak is found at 194.0 eV (d), which can be assigned to B–OH bonds. The appearance of B–O coordination at this energy is consistent with other reports in the literature.^59–63^ The local coordination environment around B at 192.8 eV in B-CN (5BA) may be due to B–N or N–B–O coordinations, such as BN_2_O or BNO_2_. The bonding environment of C and N, as probed in the C K-edge and N K-edge spectra, appears consistent between different areas/pixels of CN and B-CN (5BA), and differs from the melamine reference sample. These results indicate that (i) the precursor has mostly reacted during synthesis, and (ii) B integration has not significantly affected the core CN structure (Figure 3f,g). In the C K-edge spectrum, the sharpest peak at 288.2 eV (f) can be assigned to the C 1*s* → π* (N–C–N) transition in both CN and B-CN (5BA), while both samples may also contain surface C–C (e) and C–O bonds (shown with * in the range 291.0–292.0 eV)^64,65^ (Figure 3f). In the N K-edge spectrum, the main peaks appearing for CN and B-CN (5BA) are found at 400.0 eV (g) (N 1*s* → π* (N–C–N)) and at 403 eV (h) (assigned to either N 1*s* → π* (N–C) or interlayer π–π* interactions)^64–66^ (Figure 3g). Overall, we make two observations. First, B functionalization at the nm–μm scale is inhomogeneous (B is not found in every particle). Second, different B chemical bonds, which may point to the formation of N–B–O coordination, are present in different particles. The EELS results confirm the existence of different B chemical environments, but consistent C and N bonding environments as observed via the other characterization techniques. These findings, however, differ slightly from those of the other analyses discussed above, which characterized the bulk sample rather than individual particles and suggested only B–O/B–O–H bonding. It therefore seems likely that the schematic structures proposed (Figure 9) may contain bonding motifs that are not present in every particle of the B-CN materials. Indeed, these materials may be quite chemically and structurally inhomogeneous at the particle scale.

**Figure 10:**
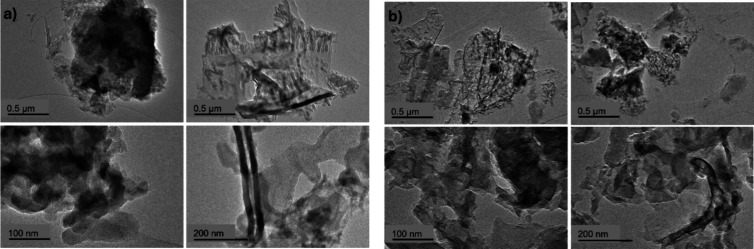
(a) TEM images of CN, and (b) TEM images of B-CN (3B).

### 3.2 CO_2_ Adsorption

CO_2_ adsorption is the first step to CO_2_ photoreduction and, thus, characterization of the adsorptive properties is necessary for any materials of interest for photocatalytic applications. To determine the effect of B functionalization on the CO_2_ adsorption capacity, we measured the CO_2_ adsorption of the materials at 298 K and up to 1 bar (Figure 2d). Almost all samples show minimal CO_2_ uptake in these conditions (0.02–0.04 mmol g^–1^ at 1 bar). The B-CN (11BA) sample (which has the highest B content and porosity) shows an almost tenfold increase in uptake (0.20 mmol g^–1^ at 1 bar). We attribute the enhanced CO_2_ adsorption capacity to the enhanced microporosity of the sample (Table 1). To examine the CO_2_ adsorption mechanism, we measured the CO_2_ adsorption of our materials at three different temperatures (288, 298, and 308 K) up to 1 bar (Figure S10). The experimental isotherm data points (Figure S10) are fitted using the dual-site Langmuir (DSL) model, which provides a good fit. For the first time, we calculated the isosteric heat of adsorption over varying CO_2_ loadings (Table 1 and Figure S11). This was done using the Clausius–Clapeyron equation and by applying the DSL coefficients (Table S4) derived from the experimental CO_2_ adsorption data (Figure S10). The CO_2_ heat of adsorption of the materials remains constant with increasing CO_2_ loading and does not vary significantly with B functionalization (11.4–17.8 kJ mol^–1^ at zero loading). Hence, the primary CO_2_ adsorption mechanism for the majority of our materials is physisorption. We cannot fully exclude though that CO_2_ could also form localized chemical bonds (chemisorption) with −NH_2_ groups in the structure, as suggested in computational studies.^26,27,31^ An increased CO_2_ uptake in B-CN materials, compared to pristine CN, has been previously reported in the literature for thermally exfoliated B-CN nanosheets.^26^ Bulk CN-based materials usually adsorb less CO_2_ than their 2D-shaped counterparts; however, B-CN (11BA) adsorbs as much CO_2_ as ultrathin nanoporous B-CN nanosheets, and outperforms other reported bulk and shaped (in nanosheets) B-CN.^26,31^

### 3.3 Optoelectronic Properties

To probe the optoelectronic properties of our materials, we employed several techniques including DRS UV–vis spectroscopy, XPS valence band and work function measurements, APS, steady-state PL spectroscopy, and TAS (Figure 11). Initially, to examine the effect of B functionalization on light harvesting, we measured our materials by using DRS UV–vis (Figure 11a). Like CN, all B-functionalized materials exhibit two peaks at ∼250 and ∼370 nm. This bimodal absorption indicates two different electron transitions upon excitation. The band at ∼250 nm corresponds to the allowed π–π* transition from electrons in the sp^2^-hybridized bonds (C–N=C).^67,68^ The band at ∼370 nm corresponds to the allowed n−π* transition from N-lone pair electrons, or to/from chemical moieties (i.e., C=O, −NH_2_, V_N_) in defective CN.^67,68^ All materials show similar curves and absorption edges (around 450 nm), and we conclude that B functionalization does not affect light absorption significantly. However, sample B-CN (3B) exhibits significant absorbance at higher wavelength compared to the 450 nm absorption onset of CN, potentially due to the agglomeration of B atoms in the structure. Using the Kubelka–Munk function, we converted the DRS UV–vis data into Tauc plots to determine the indirect allowed bandgap transition (Figure 11b).^69^ By linear regression in the zoomed-in area (edge) of the Tauc plots (Figure 11c), we identified the bandgaps of the materials in the range 2.6–2.7 eV, which agrees well with previous reports.^8,28,31^ Band edge energies were determined using XPS valence band and work function, and APS measurements. Through XPS valence band analysis (Figure 11d), we identified the XPS valence band energy of the samples, which ranges between 2.2 and 2.4 eV. We note that these energy levels need to be added to the work functions of the materials (Figure S12) for the calculation of the true valence band position (*E*_V_) vs vacuum (*E*_vac_). Therefore, the calculated (from XPS) *E*_V_ varies from 5.6 to 6.2 eV. For comparison, we also measured the *E*_V_ of the materials using APS (Figure 11e). APS measurements performed at ambient conditions reveal somewhat deeper *E*_V_: 6.5 eV for CN, 6.7 eV for B-CN (3B), and 6.8 eV for B-CN (11BA), confirming the trend with B functionalization. We calculated the band structures of the materials vs *E*_vac_ (eV), vs normal hydrogen electrode NHE (V), and vs saturated calomel electrode SCE (V), based on the bandgaps, XPS valence band, and work function (Figure 11f). The band edge values align with literature for B-functionalized CN samples.^8,28,31^ While B functionalization does not alter the light absorption (bandgap), it shifts the band edges to deeper energy levels and brings the Fermi level (*E*_F_) closer to the conduction band (*E*_C_), increasing n-type semiconductor behavior. However, due to the decrease in the conduction band reduction potential, this modification can be problematic for photocatalytic CO_2_ reduction. Indeed, the CO_2_ activation potential (−1.9 V vs NHE) is higher than the conduction band potential of the materials (max −1.5 V vs NHE), especially for the B-CN samples.

**Figure 11:**
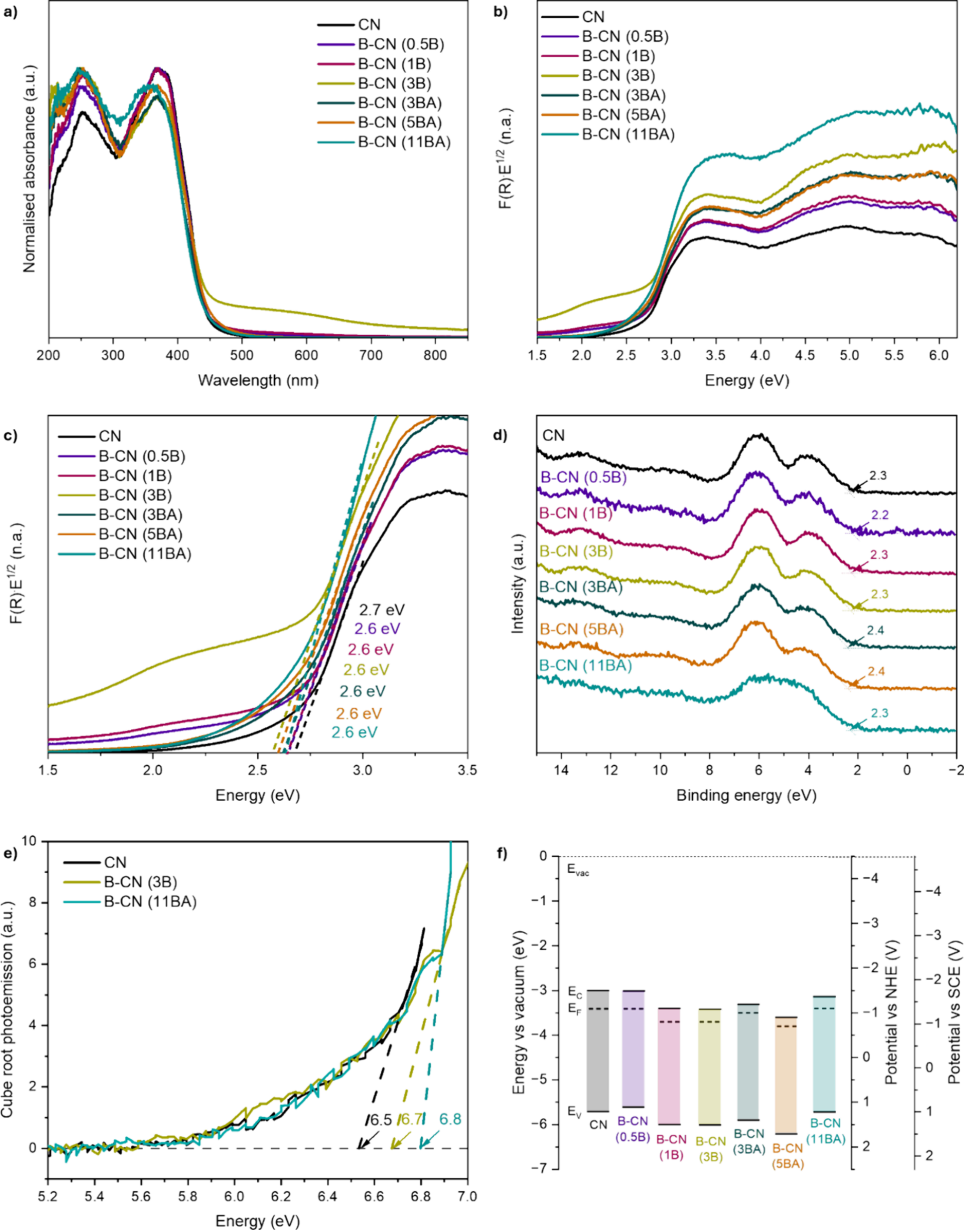
Spectroscopic analyses of the pristine and B-functionalized CN samples to analyze optoelectronic properties: (a) normalized DRS UV–vis spectra, (b, c) Tauc plots and zoomed-in area of Tauc plots, (d) XPS valence band spectra, (e) APS valence band spectra of CN, B-CN (3B) and B-CN (11BA), and (f) electronic band structures vs vacuum, vs NHE, and vs SCE.

To study the pattern of light emission upon charge carrier recombination, we measured the steady-state PL of the selected materials: CN, B-CN (1B), B-CN (5BA), and B-CN (11BA) (Figure 12a). There is a drop in PL intensity with an increasing amount of B functionalization, following the trend: CN > B-CN (1B) > B-CN (5BA) > B-CN (11BA). This gradual decrease of PL intensity could indicate two different and non-mutually exclusive phenomena. First, it could be due to a gradual decrease in the electron–hole (e^–^-h^+^) radiative recombination rate, hence the reduced light emission. Second, it could also come from an increased propensity for the nonradiative recombination pathway. Other than the drop in PL intensity, extra peaks are seen at 2.20, 2.39, 2.50, and 2.55 eV with increased B content, especially for the B-CN (BA) samples. These extra peaks point to midgap states, which may act as trapping sites for excited electrons usually right below the conduction band or right above the valence band. When we fitted the experimental steady-state PL data using the Gaussian model^70^ (Figure S13), the main PL peak for each material can be deconvoluted into two contributions. The first corresponds to the bandgap energy (2.60–2.70 eV), and the second one is approximately 0.25 eV lower than the bandgap energy (2.34–2.48 eV). Therefore, we suggest the main electron excitation states in our materials are the conduction band (emission *E*_1_ in Figure 12a), and midgap states ∼0.25 eV below the conduction band (emission *E*_2_ in Figure 12a).^70^

**Figure 12:**
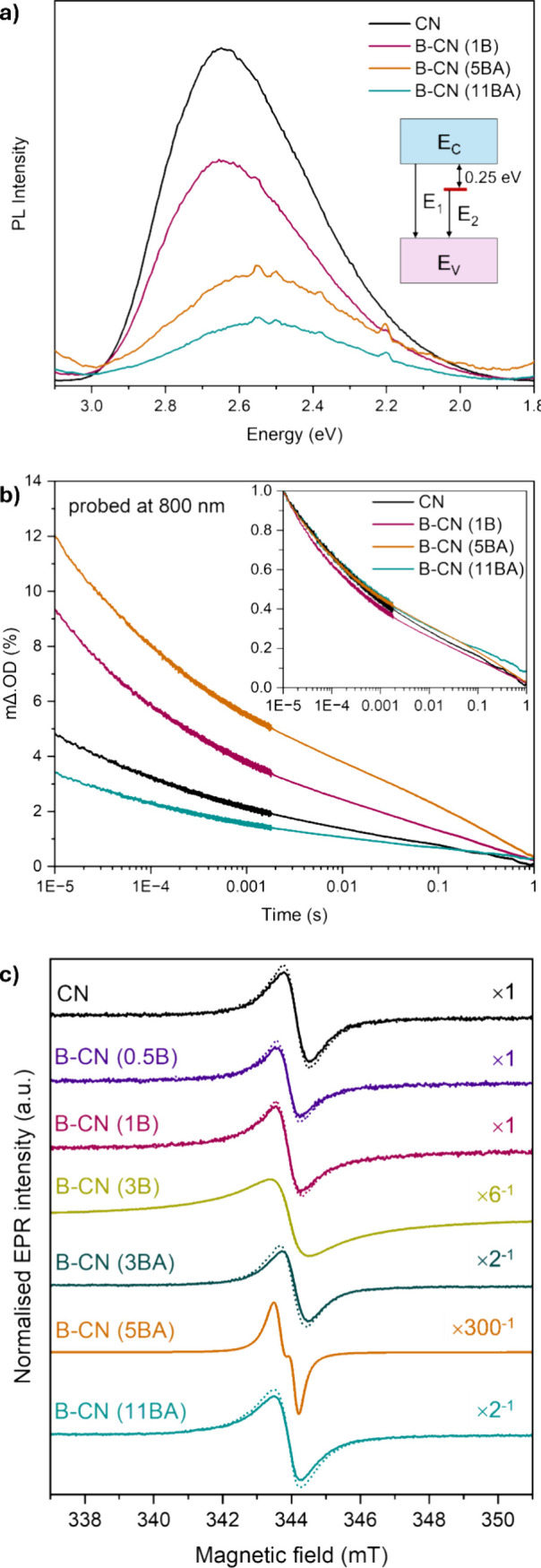
Spectroscopic analyses of the pristine and B-functionalized CN samples to analyze photoexcited species’ behavior: (a) steady-state PL spectra of CN, B-CN (1B), B-CN (5BA), and B-CN (11BA) samples, with indication of main light emissions in the inset, (b) TAS decay profiles of CN, B-CN (1B), B-CN (5BA), and B-CN (11BA) samples, with normalized profiles displayed in the inset, and (c) EPR spectra of pristine and B-functionalized CN materials at room temperature in air, before (solid line) and after (dashed line) irradiation. Multiplication factors used to normalize the spectra to a similar scale are shown in the right side of the plot in (c).

To further examine the charge carrier behavior in the B-functionalized materials, we carried out, for the first time on B-CN materials, TAS measurements in air using a 355 nm laser source (Figure 12b). We then compared the results with and without the use of scavengers (TEOA, AgNO_3_) (Figures S14–S20). Overall, the initial amount of charge carriers produced at 10 μs follows the trend: B-CN (5BA) = 12 mΔ.OD > B-CN (1B) ∼ 9 mΔ.OD > CN ∼ 5 mΔ.OD > B-CN (11BA) ∼ 3.5 mΔ.OD. Therefore, at this timescale, increasing B content (up to 5 at%) enhances the concentration of excited species, however, this is reversed at higher B content (11 at%). Looking at the normalized TAS decay profiles (inset of Figure 12b), we do not see a significant change in the charge carrier lifetimes upon B functionalization. We calculated the half-times, i.e., the time at which the initial TAS signal at 10 μs reaches 50% of its initial value. The half-times of the materials are: B-CN (11BA) = B-CN (5BA) = 590 μs > CN = 540 μs > B-CN (1B) = 340 μs. The charge carriers are long-lived, and their half-times are longer than some TiO_2_ materials (∼80 μs), other CN materials (∼60–90 μs), and heterojunctions thereof (10–90 μs), probed at the same wavelength (800 nm) with μs-TAS.^71–73^ Based on the shape of the TAS decay curve and the TAS spectra probed at different wavelengths (Figures S14–S20), the charge carriers seem to be constantly mobile, and their potential energy changes over time. Hence, we conclude that B functionalization does not significantly affect carrier kinetics. The use of TEOA (a hole scavenger) seems to have different impacts on pristine CN and B-functionalized CN samples (Figure S20). In the presence of TEOA, the amount of charge carriers produced by CN is increased, although their lifetimes are not affected. On the other hand, for B-CN samples, the amount of charge carriers is reduced, and their lifetimes are extended. AgNO_3_ (an electron scavenger) does not have the desired effect on any material, as it reduces the amount of charge carriers and does not influence their lifetimes. We are the first to characterize charge carrier behavior in B-CN materials using μs-TAS, including the effect of scavengers. Yet, a similar effect of TEOA and AgNO_3_ on the concentration of photoexcited species and lifetime has been reported previously for pristine CN.^70^ Specifically, the study showed that in pristine CN, the initial amplitude of photoexcited species on the μs scale increases with TEOA, and decreases with AgNO_3_. In addition, it was reported that charge carrier lifetimes are extended with TEOA and not affected by AgNO_3_.

As an extra level of confirmation for the creation of photoexcited electron carriers in the materials, we performed EPR measurements at room temperature in air, before and after irradiation with a light source. The EPR signal intensity is proportional to the concentration of unpaired electrons in a material. Almost all materials show an increase in EPR signal intensity upon irradiation (Figure 12c), which can be attributed to the creation of excited electrons. The electrons move from σ to π bonding (the source of the EPR signal) upon excitation, as suggested in the literature.^54^ Samples B-CN (3B) and B-CN (5BA) are the only materials that do not exhibit a difference in the EPR signal upon irradiation. Notably, these two samples show major differences in chemistry, as shown by the XPS and EPR analyses (described above), and display the highest EPR signal intensities under no illumination. This feature potentially indicates that the creation of excited electrons upon irradiation is hindered at higher defect concentration (as suggested by the higher unpaired electron concentration in dark conditions).

### 3.4 Photocatalytic CO_2_ Reduction

In general, CN-based materials are often used for photocatalytic H_2_O splitting/H_2_ production^4–13^ or photocatalytic dye/organics degradation.^14–17,19–24^ In both cases, the existing literature has shown that B functionalization of CN can boost photoactivity. A few research groups have performed CO_2_ photoreduction and reported superior photocatalytic activity of B-functionalized CN materials compared to the pristine one (Table S5).^24,26–31^ In most cases, the materials were tested in a solid–gas phase setup using H_2_O vapor as the electron and proton donor. The main products were CO (up to 40.4 μmol g^–1^ h^–1^) and CH_4_ (up to 2.2 μmol g^–1^ h^–1^) from the reduction of CO_2_, and H_2_ (up to 4.4 μmol g^–1^ h^–1^) from the competitive H_2_O splitting reaction. In one case, B-CN materials were tested in the liquid phase in an aqueous suspension, and the sole gaseous product was CH_4_ (up to 42.5 μmol g^–1^ h^–1^ under visible light).^28^ Here, we tested the pristine CN and B-CN (11BA) samples for liquid-phase batch CO_2_ photoreduction, using H_2_O as a reactant, and conducted control tests (Figure 13a,c). The only products detected in the gaseous phase were CO and H_2_—in accordance with the literature. B-CN (11BA) produces as much CO (0.9 μmol g^–1^ h^–1^) and less H_2_ (4.4 μmol g^–1^ h^–1^) compared to pristine CN (0.8 μmol of CO g^–1^ h^–1^ and 39.5 μmol of H_2_ g^–1^ h^–1^). Moreover, we notice that CO is also formed in the absence of the catalyst (∼0.014 μmol h^–1^) and when using CN with no irradiation (∼0.008 μmol h^–1^). The CO production from these control tests accounts for >80% of the CO production measured when using CN under normal reaction conditions (0.078 μmol h^–1^). The limited activity of the B-CN compounds studied here may be due to their optoelectronic properties. CN and B-CN (11BA) have similar charge carrier populations formed at the μs scale and similar charge carrier lifetimes (recombination rates) and kinetics (Figure 12b). In addition, B functionalization favorably increases the VB potential, facilitating H_2_O oxidation, but unfavorably increases the CB potential, hindering CO_2_ activation to CO_2_^–^ (if this is indeed the activation pathway) (Figure 11f). On another hand, the availability of CO_2_ molecules in an aqueous suspension is a limiting factor to the reaction, as CO_2_ solubility in pure H_2_O in ambient pressure and temperature is minimal.

**Figure 13:**
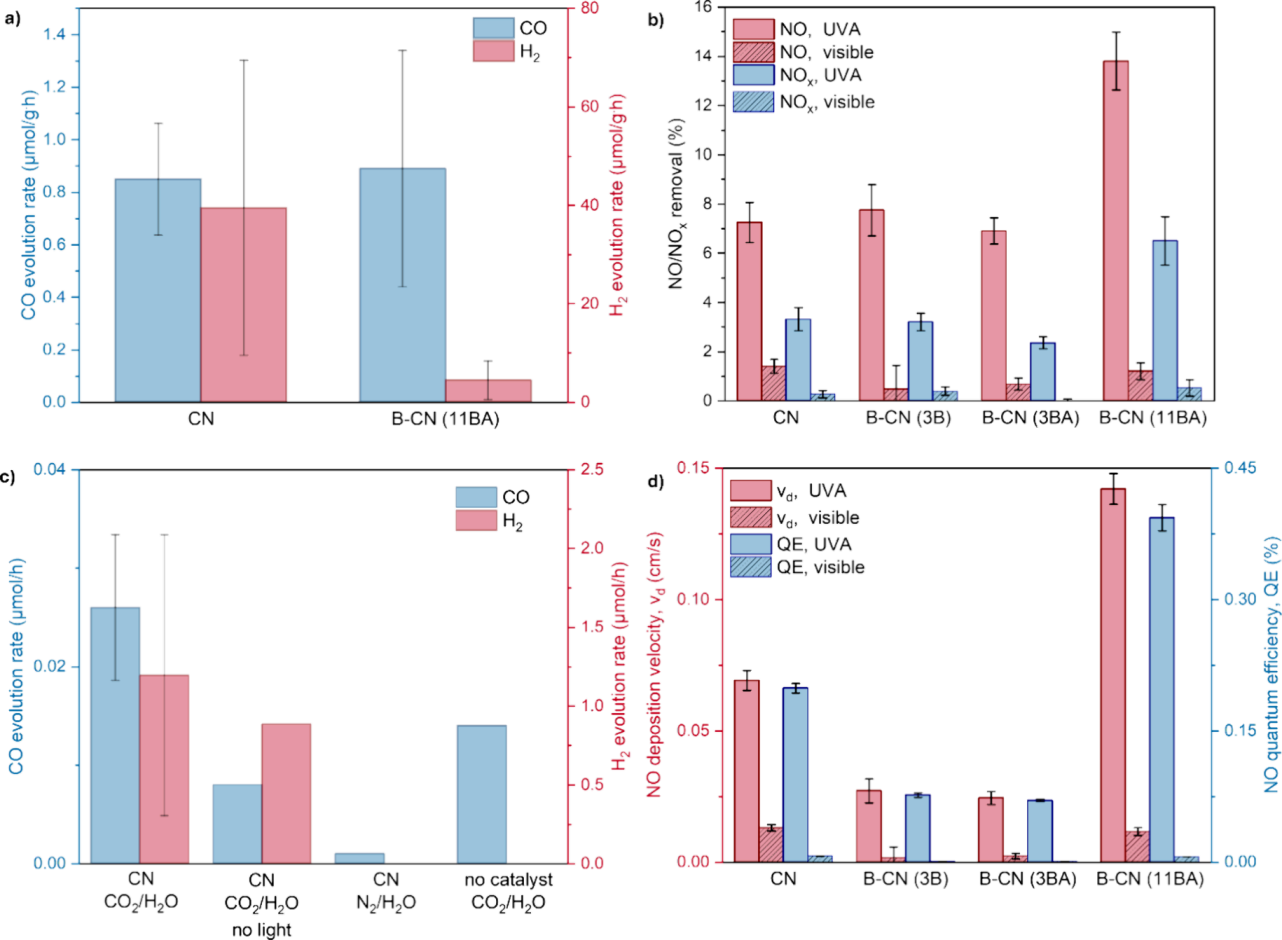
Photocatalytic CO_2_ reduction and NO_*x*_ removal results: CO and H_2_ product evolution rates for (a) pristine CN and B-CN (11BA) samples after a 5 h liquid-phase reaction under CO_2_/H_2_O, and (c) pristine CN sample after a 5 h liquid-phase reaction under CO_2_/H_2_O and control tests (under CO_2_/H_2_O without irradiation, under N_2_/H_2_O, and no catalyst under CO_2_/H_2_O). The left axis is scaled and assigned to the CO evolution rate, while the right axis is scaled and assigned to the H_2_ evolution rate. (b) NO/NO_*x*_ conversion percentage and (d) NO deposition velocities/quantum efficiencies for samples CN, B-CN (3B), B-CN (3BA), and B-CN (11BA). In (d) left axis is scaled and assigned to NO deposition velocity, while the right axis is scaled and assigned to NO quantum efficiency. The error bars in (a–d) represent 95% confidence intervals.

### 3.5 Photocatalytic NO_*x*_ Removal

Another light-assisted reaction of interest is photocatalytic NO_*x*_ (including NO/NO_2_) removal, owing to its role in combating environmental pollution and lingering public health issues. In this reaction, the aim is to achieve high NO removal from the inlet air stream, whilst limiting the conversion of NO to toxic NO_2_ (i.e., low NO_2_ selectivity), thus favoring conversion to nontoxic NO_3_^–^.^74^ We tested the CN, B-CN (3B), B-CN (3BA), and B-CN (11BA) materials for photocatalytic NO_*x*_ removal according to the ISO BS 22197–1:2016 protocol, to investigate the effects of B functionalization, the choice of B precursor, and B content (Figure 13b,d). Almost all materials show similar NO conversion percentages (Figure 13b) under UVA light (6.9–7.8%). An exception is B-CN (11BA), whose conversion percentage is twice that of pristine CN and other B-CN samples (13.8%). The removal of NO under visible light is minimal (<2%) for all materials, with CN and B-CN (11BA) showing the best performance (1.4 and 1.2%, respectively). As we mentioned, NO can be converted into NO_2_ (undesired) and NO_3_^–^ (desired). NO_*x*_ removal refers to the removal of both NO and produced NO_2_ and is a direct measure of the efficiency of a photocatalyst, and an indirect measure of the selectivity to NO_2_. Total NO_*x*_ removal under UVA light follows the trend: B-CN (11BA) (6.5%) > CN (3.3%) > B-CN (3B) (3.2%) > B-CN (3BA) (2.4%). Total NO_*x*_ removal under visible light is nominal, with B-CN (11BA) showing the highest performance (0.5%). NO_2_ selectivity under UVA light follows the trend: B-CN (3BA) (64.9%) > CN (53.0%) > B-CN (11BA) (51.7%) > B-CN (3B) (26.2%). We also calculated the NO deposition velocity and quantum efficiency for our materials (Figure 13d). The NO deposition velocity/quantum efficiency under UVA light follows the same trend, where B-CN (11BA) (0.14 cm s^–1^/0.39%) > CN (0.07 cm s^–1^/0.20%) > B-CN (3B) (0.03 cm s^–1^/0.08%) > B-CN (3BA) (0.02 cm s^–1^/0.07%). For photocatalytic NO_*x*_ conversion, the generally accepted mechanism relies on O_2_ reduction to form superoxide, and both pristine CN and B-CN have large overpotentials to drive this reduction reaction. For H_2_O oxidation to hydroxyl radicals, B-CN will have a more favorable kinetic overpotential to drive this reaction, compared to pristine CN, resulting in greater activity with high B functionalization. To our knowledge, the use of B-functionalized CN materials has been studied twice before for photocatalytic NO_*x*_ removal (Table S5).^24,32^ Wang et al. compared the NO conversion and NO_2_ selectivity percentages over bulk CN, CN hollow nanotubes, and B-doped CN hollow nanotubes with different B contents (although the B content was not specified). They reported enhanced NO removal (25–30%) and low NO_2_ selectivity (24–49%) of B-CN nanotubes compared to pristine CN (nanotubes and bulk) under visible light irradiation. They also suggest a potential mechanism, where NO_*x*_ is primarily converted to NO_3_^–^ through: (i) a one-step reaction with photogenerated holes and (ii) a two-step reaction of O_2_ with photogenerated electrons to form ^·^O_2_^–^, which then reacts with NO to form NO_3_^–^.^32^ Jin et al. also presented higher NO removal under visible light irradiation (25–26%) for the B-doped samples.^24^ Overall, our selectivity percentages are in accordance with the literature, although our NO conversion percentages are lower than those reported for visible light activity. We note that the experimental conditions, sample morphology, and B content are not the same in the two cases, and therefore a direct comparison is difficult. We can summarize that the choice of B precursor does not play an important role, however, B functionalization at a high B content (11 at%) enhances photocatalytic NO_*x*_ removal and efficiency. Based on the literature, 1D CN-based structures (like nanotubes) may work better for this reaction than the bulk form. However, we encourage more extensive research to be done in this field based on our findings on the influence of B content.

## 4 Conclusions

In our study, we have successfully unraveled the influence of (i) B functionalization, (ii) B content, and (iii) the choice of B dopant on the physicochemical, morphological, adsorptive, and optoelectronic properties of bulk CN. We have explored the relationship between such properties and photoactivity through advanced characterization. More specifically, we have produced two sets of B-functionalized CN samples with varying B content (0.5–11 at%), using two B precursors: amorphous boron and boric acid. We demonstrate that B is integrated into the CN structure through B–O bonds—contrary to current reports in the literature claiming B_C_ substitution—and additionally in B clusters when using elemental boron as the precursor. Although we have shown homogeneity of B functionalization at the bulk level, heterogeneities in both morphology and elemental dispersion occur in the nm to μm scale. High B content results in increased surface area and enhanced CO_2_ adsorption, and B functionalization lowers the band edges without changing the bandgap of the material. All materials show a similar tri-s-triazine structure and light absorbance; however, they exhibit different relaxation patterns and creation of midgap states. Charge carrier lifetime and decay behavior were not significantly affected by B content, however, the concentration of charge carriers seen maximizes at 5 at%. We notice differences in the concentration of unpaired electrons, which could be linked to the chemical structure changes caused by B integration from different precursors. Most materials show a change in EPR signal intensity before and after irradiation, an indication of the number of excited electrons. We have also tested our materials for CO_2_ photoreduction and photocatalytic NO_*x*_ removal and discovered that B functionalization alone is not enough to make bulk CN an efficient standalone photocatalyst.

Comparing the two B precursors and their impact on the final materials, we observe that the amount of functionalization is better controlled using boric acid, owing to its greater reactivity with melamine. The choice of precursor creates different chemical environments, and the B content shifts the ratio between the different B coordinations. Here, boric acid could be preferred over amorphous B, as the latter results in unreacted, agglomerated B atoms. The type of B precursor does not seem to strongly influence the bandgap, charge carrier lifetimes, and kinetics. However, at the μs scale, the initial concentration of charge carriers is affected by B content, and a moderate B content (5 at. %) is preferred. For NO_*x*_ photoremoval, only a high B content has an enhancing effect (11 at%), which could only be achieved using boric acid as the precursor.

The scope of this study was the investigation of fundamentals rather than optimization. Building on this work, a way forward to test these materials in more favorable conditions would be to synthesize CN-based heterojunctions. Another way would be to add cocatalysts to overcome the CO_2_ activation barrier and use H_2_O–CH_3_OH or H_2_O-TEOA solutions for CO_2_ saturation. Previous literature links the enhanced CO_2_ photoreduction activity of B-CN materials to their (i) extended visible light absorption, (ii) better charge carrier separation and limited recombination rates, or (iii) higher surface area and CO_2_ uptake, compared to pristine CN. However, our findings and data do not support these claims. Overall, we encourage further research in the field, especially in the use of B-CN as a part of heterojunction systems. In such systems, each redox half-reaction of the photocatalytic process can be driven by each component while improving charge carrier separation. An additional line of research could be the addition of cocatalysts in B-CN-based heterojunctions. This could increase the kinetics of photocatalytic processes relative to the intrinsic recombination rate in B-CN.

## Supplementary Material



## Notes

Details of characterization methods (for chemical, structural and optoelectronic properties), computational methods (DFT calculations), photoreactor setup schematics, XPS core-level spectra, EPR spectra at room temperature in air, ^13^C, ^1^H, and ^11^B NMR measurements and parameters, CN models used for DFT calculations, CO_2_ adsorption isotherms, fittings and fitting coefficients, heat of adsorption, deconvoluted PL spectra, XPS work function spectra, TAS decay kinetics and spectra, and comparison of photocatalytic activity of B-functionalized gC_3_N_4_ between literature and our current work (PDF)

## ∇

Centre for Energy Research, Institute of Technical Physics and Materials Science, 1121 Budapest, Hungary
