# Supplementary material for: Boron-Functionalized Graphitic Carbon Nitride Materials for Photocatalytic Applications: Effects on Chemical, Adsorptive, Optoelectronic, and Photocatalytic Properties
Source: ACS Mater Au. 2025 May 12;5(4):656–74. doi: 10.1021/acsmaterialsau.5c00007 (PMC12257377; doi:10.1021/acsmaterialsau.5c00007)
Supplement: Supplementary file 1 [file mg5c00007_si_001.pdf]

Boron-functionalized graphitic carbon nitride materials for photocatalytic applications: effects on chemical, adsorptive, optoelectronic and photocatalytic properties

Ioanna Itskou[1], Sharminaz C. Sageer[2], Daniel M. Dawson[2], Andreas Kafizas[3,4], Irena Nevjestic[4,5], Catriona M. McGilvery[5], Matyas Daboczi[6‡], Gwilherm Kerherve[5], Salvador Eslava[6], Sandrine Heutz[4,5], Sharon E. Ashbrook[*2], Camille Petit[*1]

[1] Barrer Centre, Department of Chemical Engineering, Imperial College London, London SW7 2AZ, UK

[2] School of Chemistry, EaStCHEM and Centre of Magnetic Resonance, University of St. Andrews, St. Andrews KY16 9ST, UK

[3] Department of Chemistry, Molecular Sciences Research Hub, Imperial College London, London W12 7TA, UK

[4] London Centre for Nanotechnology, Imperial College London, London SW7 2AZ, UK

[5] Department of Materials, Imperial College London, London SW7 2AZ, UK

[6] Department of Chemical Engineering and Centre for Processable Electronics, Imperial College London, London SW7 2AZ, UK

‡ Current address: Centre for Energy Research, Institute of Technical Physics and Materials Science, Budapest 1121, Hungary

* Corresponding authors: Sharon E. Ashbrook (sema@st-andrews.ac.uk); Camille Petit (camille.petit@imperial.ac.uk)

Materials Characterization

Chemical and structural properties

For the FTIR measurements, a Cary 630 FTIR spectrometer (Agilent) equipped with an attenuated total reflectance (ATR) accessory was used. Samples were manually ground with an agate mortar and pestle and the spectra were collected after 32 repetitions per sample in the 400-4000 cm^{-1} range, with a 2 cm^{-1} resolution.

For the XPS measurements, a high-throughput KAlpha X-ray Photoelectron Spectrometer (Thermo Scientific) equipped with a monochromatic Al Ka source (hn = 1486.6 eV) was used. Samples were manually ground with an agate mortar and pestle and mounted on the XPS holder using conductive carbon tape. The X-ray power gun was set to 72 W and the measurements were performed under high-vacuum conditions (~10^{-11}-10^{-10} bar) with the use of a flood gun to prevent sample charging. Measurements were taken at three different points/areas of the mounted sample, and averaged. Data analysis on the survey scans and core level (B 1*s*, C 1*s*, N 1*s*, O 1*s*) spectra was performed using the Thermo Avantage software. The adventitious carbon (C-C) peak at 284.8 eV was used for binding energy calibration.

EPR measurements were performed using an Elexsys E500T continuous-wave CW EPR spectrometer (Bruker) operating at X-band frequencies (9.5–9.9 GHz/0.35 T) and equipped with an ER4118-X MD5 resonator (Bruker). The microwave frequency used was 9.63 GHz with a microwave power of 2 mW, 100 kHz modulation frequency with 2 G modulation amplitude. The samples were placed inside 4 mm EPR quartz tubes, and all spectra were recorded at room temperature in air. Each EPR tube was filled with the sample to the same height. EPR spectra are normalised for the mass of the samples. Care was taken to position the centre of each sample in the centre of the resonator.

Solid-state NMR spectra were acquired using Bruker Avance III spectrometers equipped with either a 14.1 T or 9.4 T wide-bore magnets. Powdered samples were packed into ZrO_2 rotors and rotated at a magic-angle spinning (MAS) rate of 14 kHz using a 4-mm HFX boron-free (14.1 T) or 4-mm HFXY (9.4 T) probe. ^{11}B NMR spectra were acquired at a Larmor frequency of 192.6 MHz. Spectra were acquired using either i) a single pulse with a short flip angle (b = p/12, n_1 = 125 kHz) and recycle intervals of 1 s and 5 s for samples prepared using B and BA precursors, respectively, or ii) a spin echo experiment (also with short flip angle pulses) and a recycle interval of 0.5 s. Multiple quantum (MQ) MAS[1] experiments were carried out using a triple-quantum z-filtered pulse sequence[2] (rotor-synchronised where possible). Spectra are shown after a shearing transformation and are referenced in the indirect dimension according to the convention of Pike *et al.*[3]. Chemical shifts are quoted in ppm relative to $BF_3.Et_2O$ in $CDCl_3$ measured using a secondary reference of BPO_4 (d_{iso} = −3.3 ppm).^{1}H NMR spectra were acquired at a Larmor frequency of 400.13 MHz. Spectra were acquired using the DEPTH pulse sequence to remove probe background, a 90° pulse (n_1 = 80-100 kHz) and a recycle interval of 3 s. Chemical shifts are quoted in ppm relative to $Si(Me)_4$ in $CDCl_3$ measured using a secondary reference of L-alanine ($d_{iso}(CH_3)$ = 1.3 ppm). ^{13}C NMR spectra were acquired at a Larmor frequency of 100.6 MHz. Spectra were acquired using either i) cross polarisation (CP) with a contact pulse (ramped for ^{1}H) of 7.5 ms, SPINAL-64[4] decoupling (n_1 = 90 kHz) and a recycle interval of 3 s or ii) a spin echo (n_1 = 125 kHz) with a recycle interval of 120 s. Chemical shifts are quoted in ppm relative to $Si(Me)_4$ in $CDCl_3$ measured using a secondary reference of L-alanine ($d_{iso}(CH_3)$ = 20.5 ppm).

Samples were prepared for TEM and EELS by suspending the powders in ethanol and drop-casting onto lacey (EELS) or holey (TEM) carbon grids (300 mesh Cu, TAAB) after sonicating. For EELS measurements, to remove any residual hydrocarbon contaminants, the lacey grids were heated in a clean vacuum oven at 120 °C for 12 hours and then again after addition of the

sample. TEM measurements were performed using a TEM-2100Plus (JEOL) microscope with LaB_6 filament, operated at 200 kV. Images were acquired in bright-field TEM mode. EELS experiments were carried out at 300 kV on a Spectra 300 TEM (ThermoFisher Scientific) equipped with monochromator and Cs probe corrector. EELS spectra were acquired on a K3 direct electron detector mounted on a Gatan Continuum K3 HR spectrometer (Ametek). The convergence and collection semi angles were 15.7 mrad and 30.3 mrad, respectively, with a probe current of around 40 pA as measured on the fluscreen to minimise electron beam damage. Low-loss and core-loss spectra of B, C and N K-edges were acquired in dual EELS mode with a full width at half maximum (FWHM) of the zero loss peak of 1.1 eV and sub-pixel scanning used depending on the experiment. All spectra were aligned to the zero-loss peak. The core-loss spectrum image (SI) of CN was denoised using the principal component analysis (PCA) algorithm as implemented in Gatan Microscopy Suite (Ametek). Six to thirteen components were used depending on the SI, and care was taken to ensure no obvious artifacts from the PCA were present in the data in the regions being studied. Background subtraction of the B, C and N K-edges was then carried out using the power law method.

Powder XRD data were collected using an X'Pert Pro X-ray diffractometer (PANalytical) with an anode voltage of 40 kV and an emission current of 40 mA using monochromatic Cu Ka_1 (l = 1.54060 Å) and Cu Ka_2 (l = 1.54443 Å) radiation, emitted with an intensity ratio of 2:1. The XRD detector used was an X'Celerator silicon strip detector. Points were recorded over a 2q angle range of 5 to 65° with a step size of 0.0167°.

N_2 (77 K) adsorption-desorption isotherms were obtained using a 3Flex Porosity Analyzer (Micromeritics). Prior to the measurements, the samples were degassed ex-situ using a VacPrep Degasser (Micromeritics) at 393 K overnight at 2×10^{-5} bar. The samples were then degassed in-situ at 393 K for 4 h down to 7×10^{-5} bar using the 3Flex Porosity Analyzer. The specific surface areas were calculated using the Brunauer-Emmett-Teller (BET) method[5]. The total pore

volume was estimated from the amount of adsorbed N_2 at $P/P_0 = 0.99$. The micropore volume was determined using the Dubinin-Radushkevich method[6].

Optoelectronic properties

For the DRS UV-Vis measurements, a UV/Vis-IRS-2600Plus (Shimadzu) spectrophotometer was used, equipped with an integrated sphere attachment. The resolution was set to 2 nm, with pressed $BaSO_4$ powder as a reference diffuse reflectance material. The samples were loaded and pressed into the holder until they covered the entire surface. Absorbance spectra were derived using the Kubelka-Munk function[7,8]: $F(R) = (1 - R)^2(2R)^{-1}$. The bandgaps of the materials were derived using the Tauc plot[9] ($F(R)hn^{1/n}$ *vs.* energy), extrapolating the onset linear region of the curve, with n = 2 as CN materials are indirect semiconductors[10].

XPS valence band and work function measurements were obtained using the same X-ray Photoelectron Spectrometer and processing methodology as described above. Valence band spectra were collected using 15 eV pass energy and 0.050 eV step size, with the "area" mode, which averages four points. The work functions for the materials, used for the determination of Fermi levels (E_F), were measured and averaged at five points. The work functions were determined by measuring the secondary electron cutoff in the low kinetic energy region, using gold as reference material. A sample bias of −29.47 V was applied using an ion gun, and the cutoff spectra were obtained using 10 eV pass energy and 0.100 eV step size.

APS measurements were conducted using an APS02 system (KP Technology). Thin, homogenous films of the powder samples were prepared on tin oxide-coated glass substrates. The measurements involved scanning the monochromatic UV light irradiation energy from 5.2 to 7.0 eV. The cube root of the photoemission as function of irradiation energy was plotted and extrapolated to zero to determine the valence band edge values (E_v) of the thin layers.

Steady-state PL spectra were measured at room temperature using a Cary Eclipse fluorescence spectrometer (Agilent). The excitation wavelength was set to 355 nm, with 2.5 nm excitation/emission slits, 0.5 s dwell time, 1 nm data interval, and 800 V photomultiplier tube voltage.

TAS was measured using a home-built setup in diffuse reflection mode. A Nd:YAG laser (OPOTEK Opolette 355 II, 7 ns pulse width) was used to generate 355 nm excitation pulses of 100 μJ cm^{-2}. A broadband probe light was generated from a quartz halogen lamp (Bentham IL1) and long pass filters were placed before the samples to reduce short wavelength irradiation of the sample. The light was collected in diffuse reflectance mode by a 2-inch diameter, 2-inch focal length lens and relayed to a monochromator to select the probe wavelength. A long pass filter was positioned at the entrance of the monochromator to block scattered laser light. The collected light was focused onto a Si photodiode detector (Hamamatsu S3071). Sub-ms data were processed by an electronic amplifier (Costronics) and recorded on an oscilloscope. Data on the ms timescale were simultaneously recorded by a DAQ card (National Instruments). Acquisitions were triggered by scatter from the laser excitation measured by a photodiode (Thorlabs DET10 A). A minimum of 200 laser pulses were averaged and processed using LabVIEW home-built software. The measurements were performed in air: i) on the powder samples, ii) on the powder samples in the presence of 10 vol% triethanolamine (TEOA), and iii) on the powder samples in the presence of 10 mM $AgNO_3$, all pressed between two microscope glass slides.

EPR measurements during irradiation were performed immediately after EPR measurement under dark was obtained for each sample, using the same equipment and methodology as described above. Irradiation measurements of the samples were carried out under direct illumination of an LSH302 light source (LOT Quantum Design) equipped with a 300 W Xe arc lamp (UXL-302-O, Ushio) through an optical access port.

Computational Methods

Periodic planewave DFT calculations were carried out using the CASTEP code (version 22)[11,12], using the PBE exchange correlation functional[13] and the D3-BJ semi-empirical dispersion correction scheme[14]. Core-valence interactions were described by ultrasoft pseudopotentials, accounting for scalar relativistic effects using the zeroth order relativistic approximation (ZORA[15]). Planewave energy cutoffs of 60 Ry were used for geometry optimization and NMR calculations, with the first Brillouin zone sampled using a Monkhorst-Pack grid[16] with a reciprocal space grid spacing of 0.04 2p $Å^{-1}$ in all cases. Initial structural models were obtained from Gracia & Kroll and Jürgens *et al.*[17,18]. In the geometry optimization all atomic coordinates and unit cell parameters were allowed to vary.

NMR parameters were calculated using the gauge-including projector augmented wave (GIPAW[11]) approach to reconstruct the all-electron wavefunction in the presence of a magnetic field. Calculations provide the absolute shielding tensor and electric field gradient tensor (**V**). Diagonalization provides their respective principal components, where $s_{11} \leq s_{22} \leq s_{33}$ and $|V_{xx}| \leq |V_{yy}| \leq |V_{zz}|$. The isotropic shielding, $s_{iso} = (1/3)\mathrm{Tr}(s)$, and the predicted isotropic chemical shift $d_{iso} = s_{ref} - s_{iso}$. Reference shieldings ($s_{ref}$) were determined by comparing experimental shift and calculated shielding for hexagonal BN (^{11}B, 97.4 ppm)[19] and melem (^{13}C, 175.1 ppm)[18]. The quadrupolar coupling constant, $C_Q = eQV_{ZZ}/h$ was obtained using a nuclear quadrupole moment of 4.059 fm^2 for ^{11}B[20]. From the quadrupolar magnitude, C_Q, and asymmetry, $\eta_Q = (V_{XX} - V_{YY})/V_{ZZ}$ of the quadrupolar interaction, the quadrupolar product $P_Q = C_Q (1 + \eta_Q^2/3)^{1/2}$ can be determined.

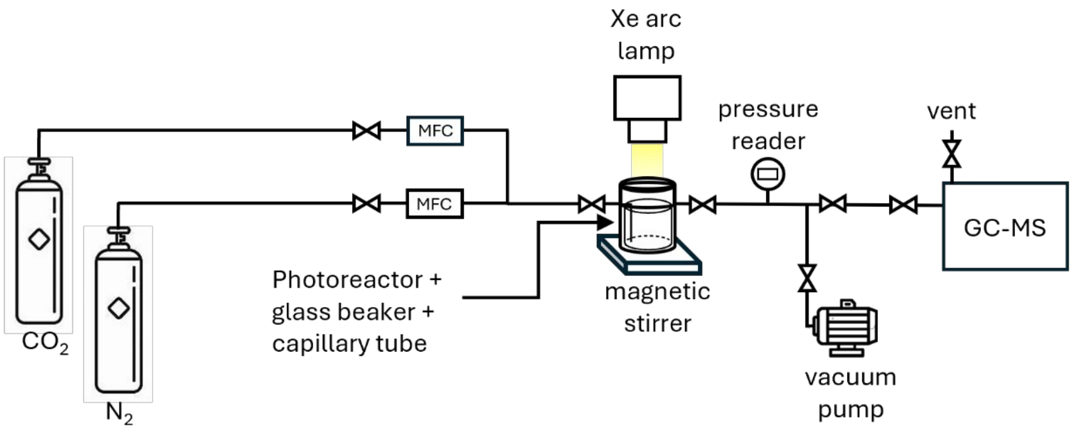

Figure S1. Schematics of the photocatalytic setup used for the CO_2 photoreduction tests in liquid phase.

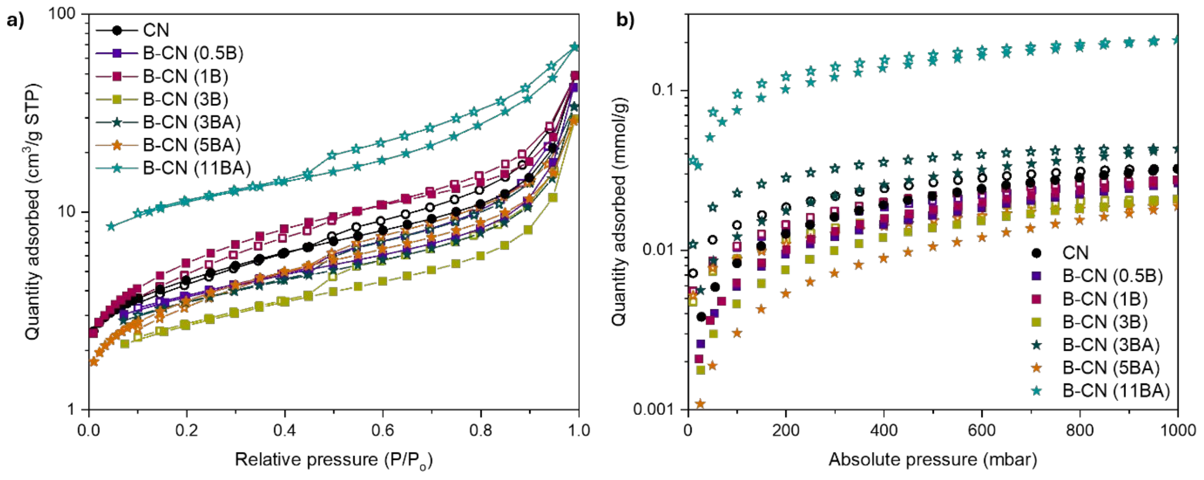

Figure S2. a) N_2 adsorption-desorption isotherms (77 K), and b) CO_2 adsorption-desorption isotherms (298 K) of pristine and B-functionalized CN samples, shown on a logarithmic scale (y-axis). Filled symbols represent adsorption and empty symbols represent desorption.

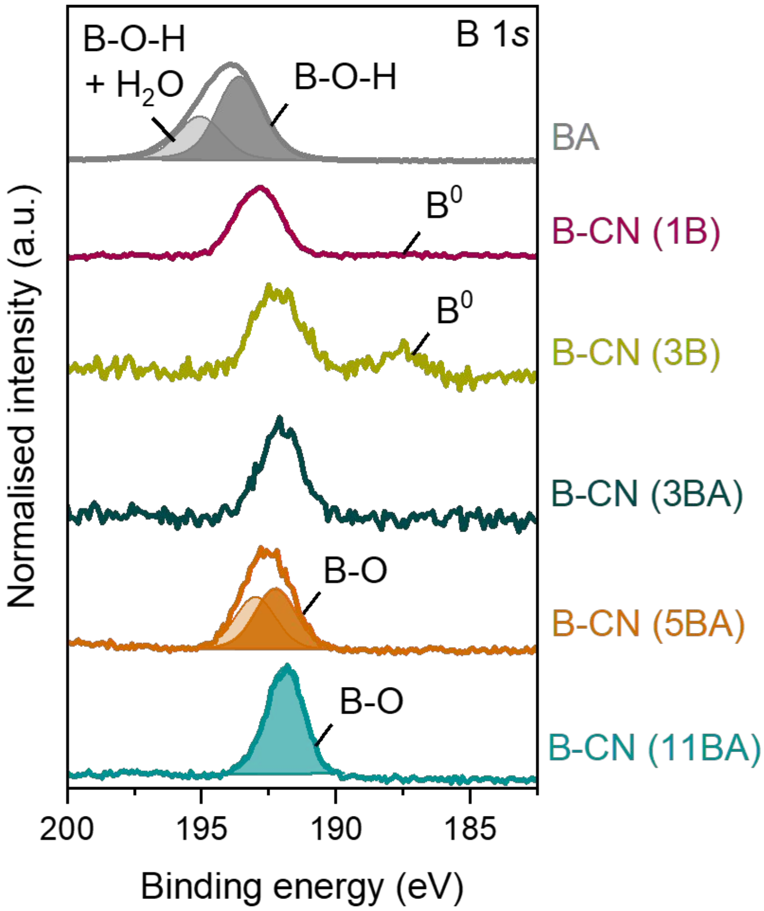

Figure S3. Expanded XPS B 1*s* spectra of B-CN (1B), B-CN (3B), B-CN (3BA), B-CN (5BA) and B-CN (11BA).

Table S1. Full width at half maximum (FWHM) values of C 1*s*, N 1*s*, B 1*s* and O 1*s* fitted peaks of B-CN (11BA) sample, before and after fitting a secondary deconvoluted B peak.

	Spectrum	Fitted peak	FWHM (eV)
	C 1*s*	C-C	1.5
		C-N, C-O	1.5
		N-C=N, C=O	1.2
	N 1*s*	C-N=C	1.2
		N-$(C)_3$	1.3
		N-H	1.3
	O 1*s*	O=C	1.7
		O-B	1.9
**Before**	B 1*s*	B-O	2.0
**After**	B 1*s*	B-O	1.8
		B-O-H (?)	1.8

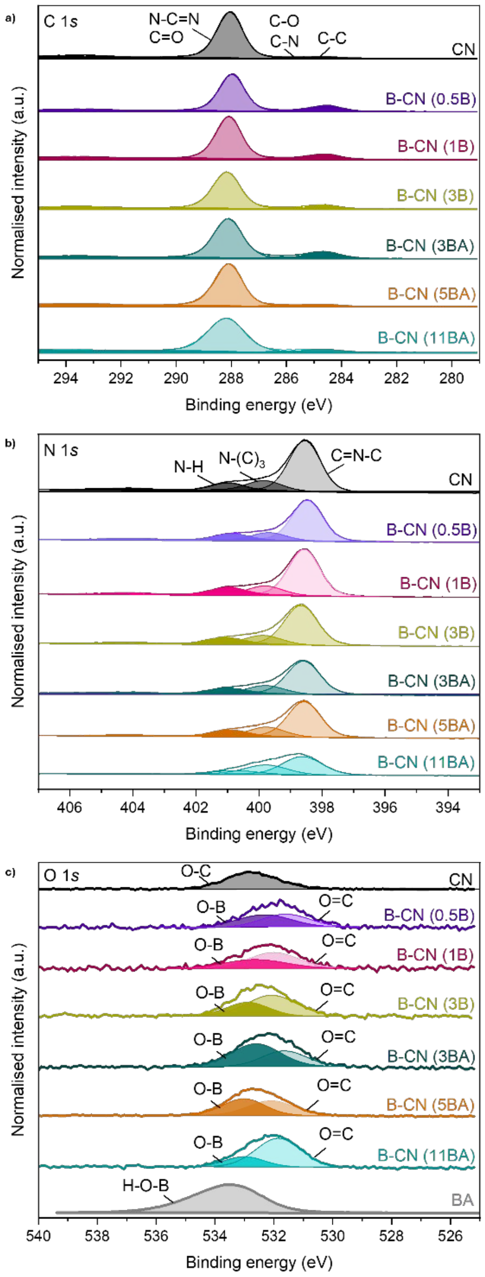

Figure S4. a) C 1*s*, b) N 1*s*, and c) O 1*s* XPS spectra of pristine CN and B-functionalized CN samples. The main bonds appearing in the C 1*s* spectra are similar between pristine and doped CN samples: N-C=N (~288 eV), C-N (~286 eV), and C-C (284.8 eV). The main peaks appearing in the N 1*s* spectra are also similar between pristine and doped CN samples: N-H (~401 eV), N-$(C)_3$ (~400 eV), and C=N-C (~398.5 eV). O 1*s* spectra change with the introduction of B-doping. The main O-C (~533 eV) peak in pristine CN shifts to a lower energy (~532 eV) for B-CN samples, which can be deconvoluted into two peaks: O-B (532.5-533 eV) and O=C (531.5-531.8 eV).

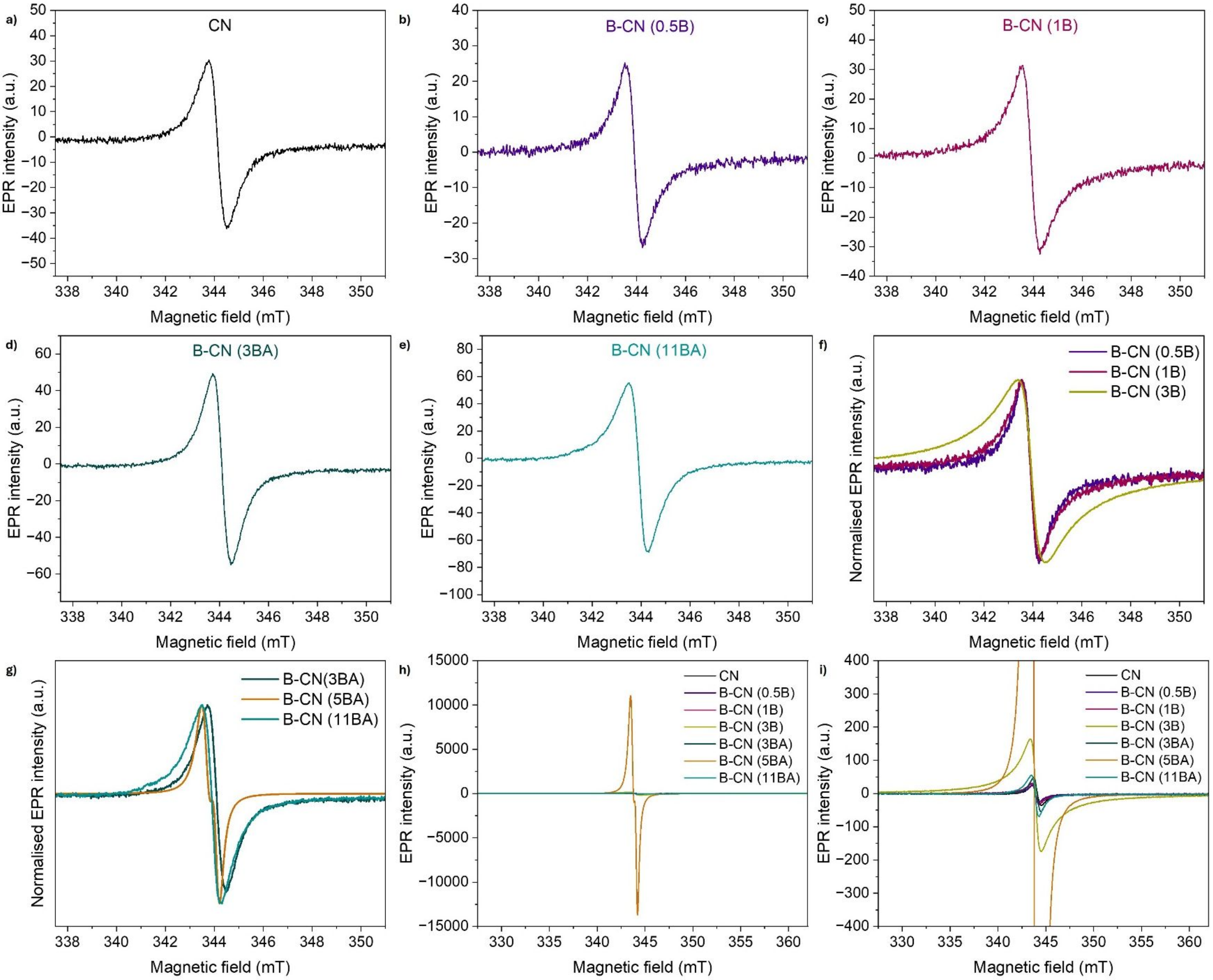

Figure S5. EPR measurements at room temperature in air of a) CN, b) B-CN (0.5B), c) B-CN (1B), d) B-CN (3BA), e) B-CN (11BA), f) B-CN (B) samples (normalized), g) B-CN (BA) samples (normalized), h) pristine CN and B-CN samples on the same scale, and i) zoomed-in pristine CN and B-CN samples on the same scale.

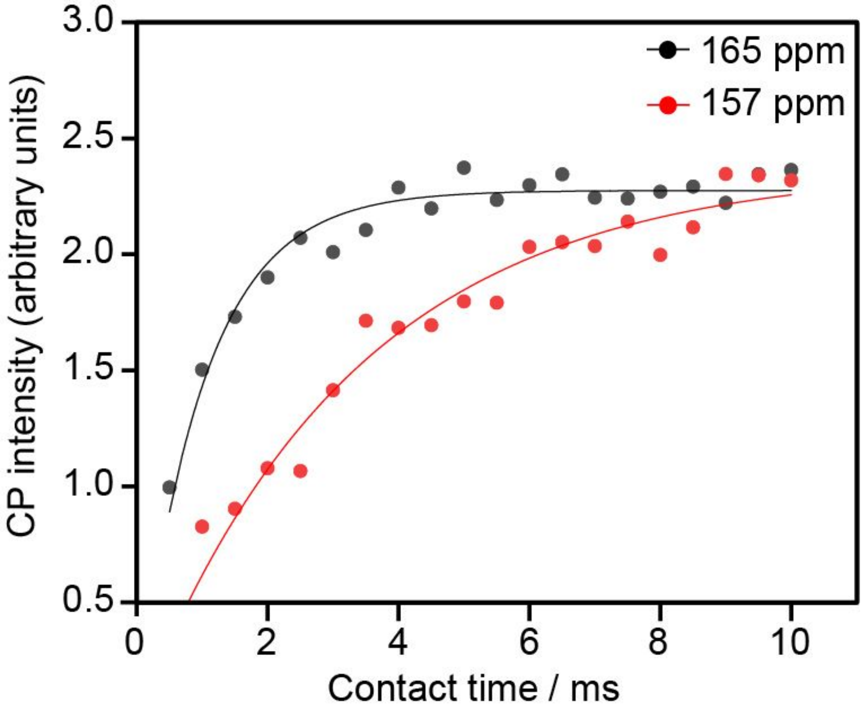

Figure S6. Plot showing the variation in the intensity of the two signals in the ^{13}C CP MAS NMR spectrum of CN as a function of contact time.

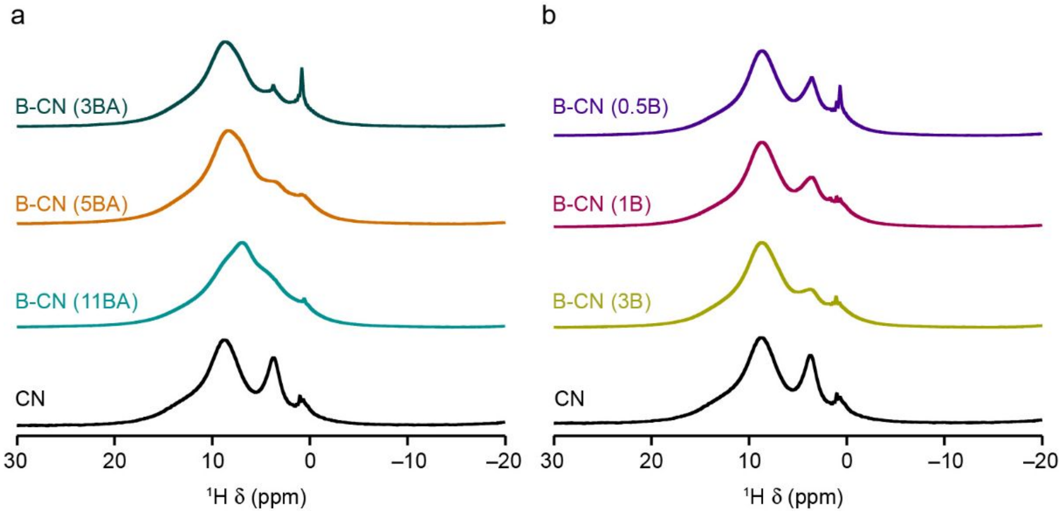

Figure S7. ^{1}H (9.4 T, 14 kHz) MAS NMR spectra acquired using DEPTH background suppression for pristine CN and a) B-CN (BA), and b) B-CN (B) samples.

Table S2. ^{13}C NMR parameters predicted using DFT for the two idealized models of CN shown in **Figure 8a** and for melem[19].

	**Ordered CN**		**Disordered CN**		**Melem ($C_6N_7(NH_2)_3$)**	
**Species**	**Type**	**δ_{iso} (ppm)**	**Type**	**δ_{iso} (ppm)**	**Type**	**δ_{iso} (ppm)**
C1	CN_3(e)	178.9	CN_3(i)	158.2	CN_3(i)	156.7
C2	CN_3(i)	161.6	CN_3(i)	162.4	CN_3(i)	157.4
C3	CN_3(i)	162.4	CN_3(e)	172.0	CN_3(i)	155.9
C4	CN_3(e)	178.4	CN_3(e)	172.6	$CN_2(NH_2)$	167.2
C5			CN_3(i)	161.4	$CN_2(NH_2)$	164.2
C6			CN_3(e)	171.8	$CN_2(NH_2)$	164.7

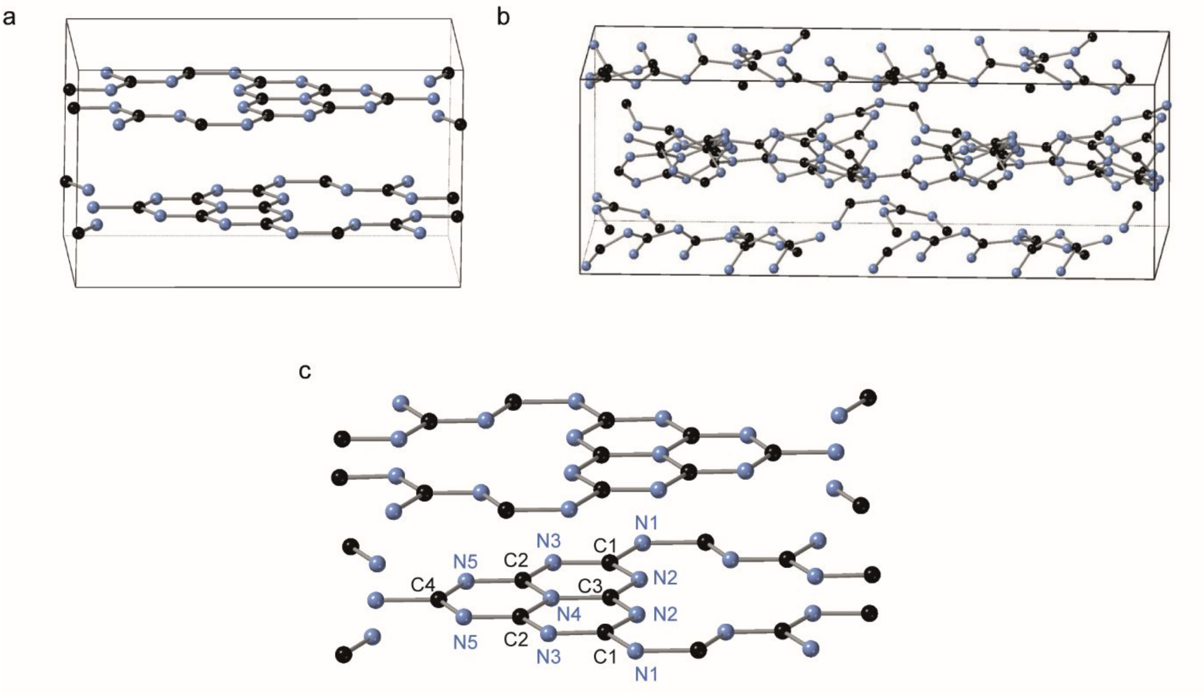

Figure S8. a) Ordered and b) disordered structural models of CN used for DFT calculations. Note there are no NH_2 groups in these idealized models. c) Numbering scheme used in the DFT calculations for the structural model in a). (carbon = black, nitrogen = blue).

Table S3. Relative energies and ^{11}B NMR parameters from DFT calculations of six models of ordered CN shown in **Figure S8a** with B functionalization.

**C site substituted for B**	**N site protonated**	**ΔΔE / eV**	**^{11}B NMR parameters**		
			**δ_{iso} (ppm)**	**C_Q / MHz**	**η_Q**
1	2	0.0885	28.9	3.14	0.41
1	3	0.8140	27.8	3.17	0.38
2	3	1.2823	24.8	2.91	0.62
2	5	0.3669	28.0	3.01	0.54
3	2	0.4776	28.0	3.02	0.55
4	5	0.0000	27.7	2.96	0.48

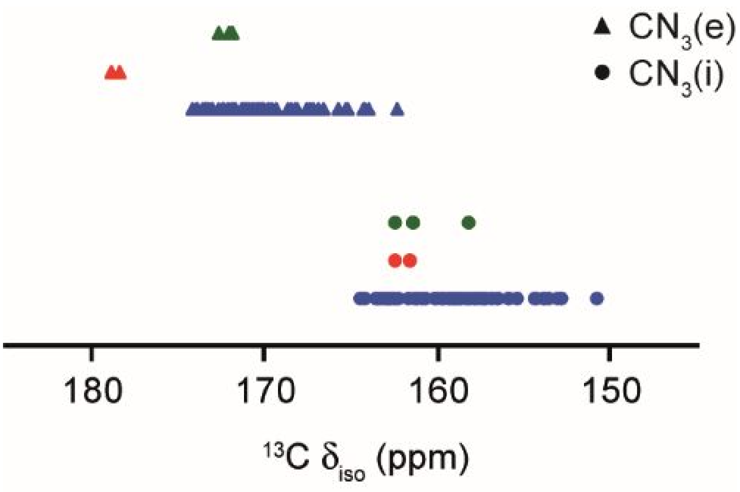

Figure S9. Calculated ^{13}C δ_{iso} from ordered (red) and disordered (green) models CN, and for B-substituted ordered CN (blue). Carbons in CN_3(e) environments are denoted with triangles and those in CN_3(i) environments with circles.

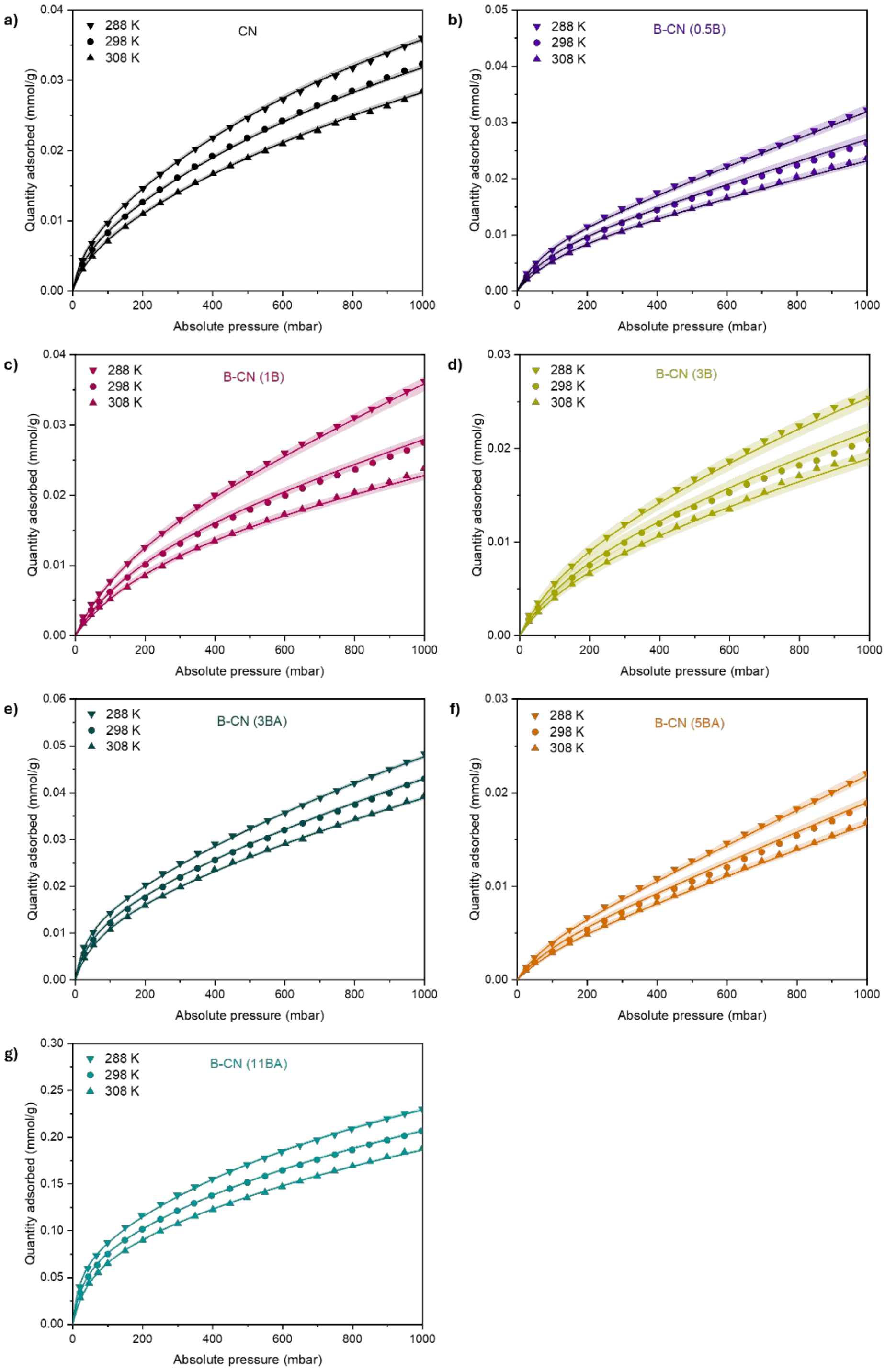

Figure S10. CO_2 adsorption isotherms measured at 288, 298 and 308 K of a) CN, b) B-CN (0.5B), c) B-CN (1B), d) B-CN (3B), e) B-CN (3BA), f) B-CN (5BA), and g) B-CN (11BA) samples. Symbols represent data points, solid lines represent dual-site Langmuir (DSL) fitting, and shading represents confidence bounds.

Table S4. Dual-site Langmuir (DSL) coefficients, as derived from fitting experimental CO_2 adsorption data for pristine and B-CN materials (**Figure S10**). Uncertainty values are presented in parentheses.

DSL coefficient	CN	B-CN (0.5B)	B-CN (1B)	B-CN (3B)	B-CN (3BA)	B-CN (5BA)	B-CN (11BA)
q_{sb} (mol kg^{-1})	0.07 (4.03·10^{-4})	8.91·10^{-3} (2.12·10^{-4})	0.02 (2.80·10^{-4})	7.78·10^{-3} (2.74·10^{-4})	0.15 (9.74·10^{-4})	3.98·10^{-3} (1.52·10^{-4})	0.07 (4.74·10^{-4})
q_{sd} (mol kg^{-1})	6.57·10^{-3} (1.07·10^{-4})	31.10 (0.46)	4.98 (0.10)	0.10 (2.16·10^{-3})	0.01 (1.44·10^{-4})	44.06 (0.56)	0.36 (1.77·10^{-3})
b_0 (bar^{-1})	6.80·10^{-4} (5.44·10^{-6})	1.22·10^{-3} (1.35·10^{-4})	0.03 (1.50·10^{-3})	0.06 (8.58·10^{-3})	1.48·10^{-3} (1.17·10^{-5})	9.97·10^{-4} (1.67·10^{-4})	7.56·10^{-5} (3.63·10^{-6})
d_0 (bar^{-1})	9.74·10^{-4} (1.15·10^{-4})	9.80·10^{-7} (1.45·10^{-8})	2.50·10^{-8} (5.00·10^{-10})	1.35·10^{-4} (3.24·10^{-6})	4.08·10^{-4} (2.88·10^{-5})	3.07·10^{-6} (3.88·10^{-8})	4.53·10^{-4} (3.15·10^{-6})
$-\Delta U_b$ (kJ mol^{-1})	16.65 (19.80)	22.26 (277.67)	12.37 (132.57)	11.40 (336.89)	12.78 (19.47)	22.43 (418.45)	31.98 (120.15)
$-\Delta U_d$ (kJ mol^{-1})	25.07 (295.77)	15.94 (36.49)	29.00 (49.07)	17.73 (59.08)	26.68 (176.08)	11.74 (31.32)	17.78 (17.21)

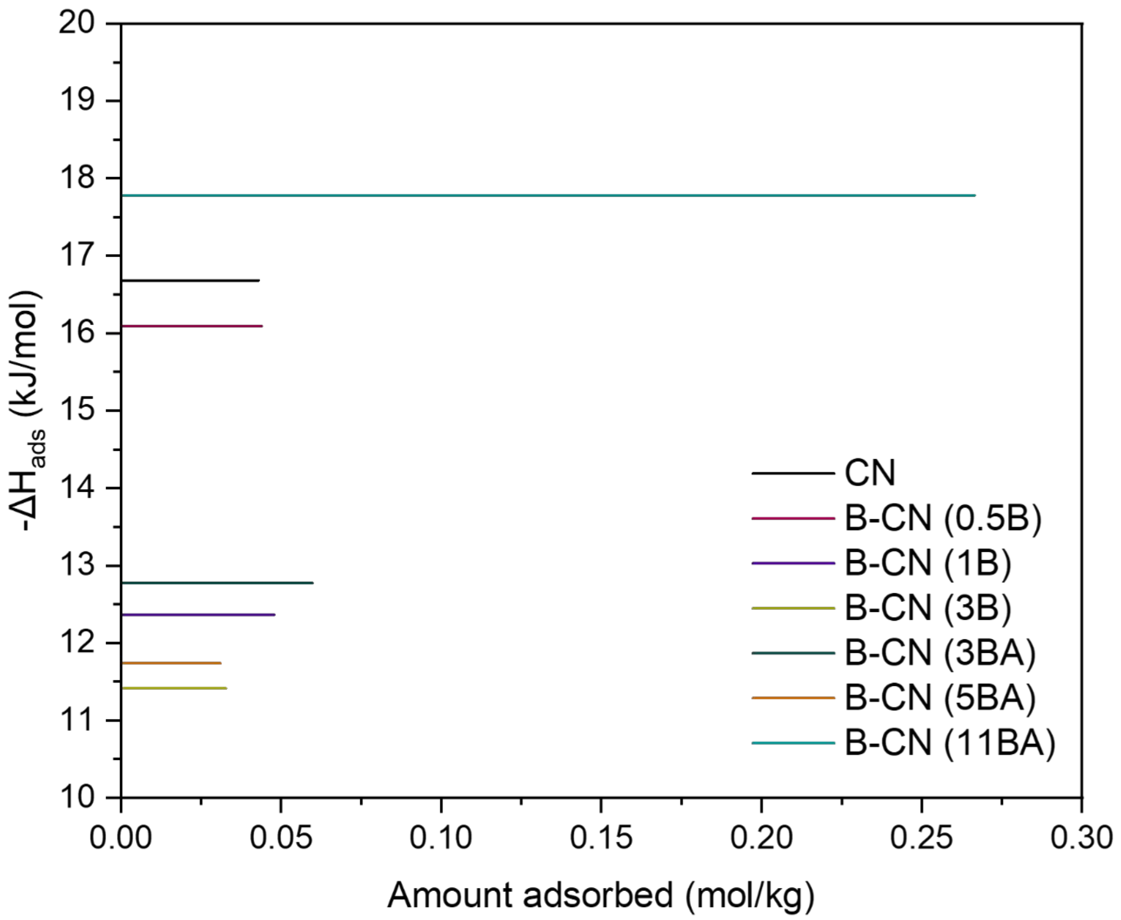

Figure S11. Isosteric heat of adsorption plotted against CO_2 loading for pristine and B-functionalized CN materials. Heat of adsorption was calculated by applying the DSL coefficients (**Table S4**) on the Clausius-Clapeyron equation.

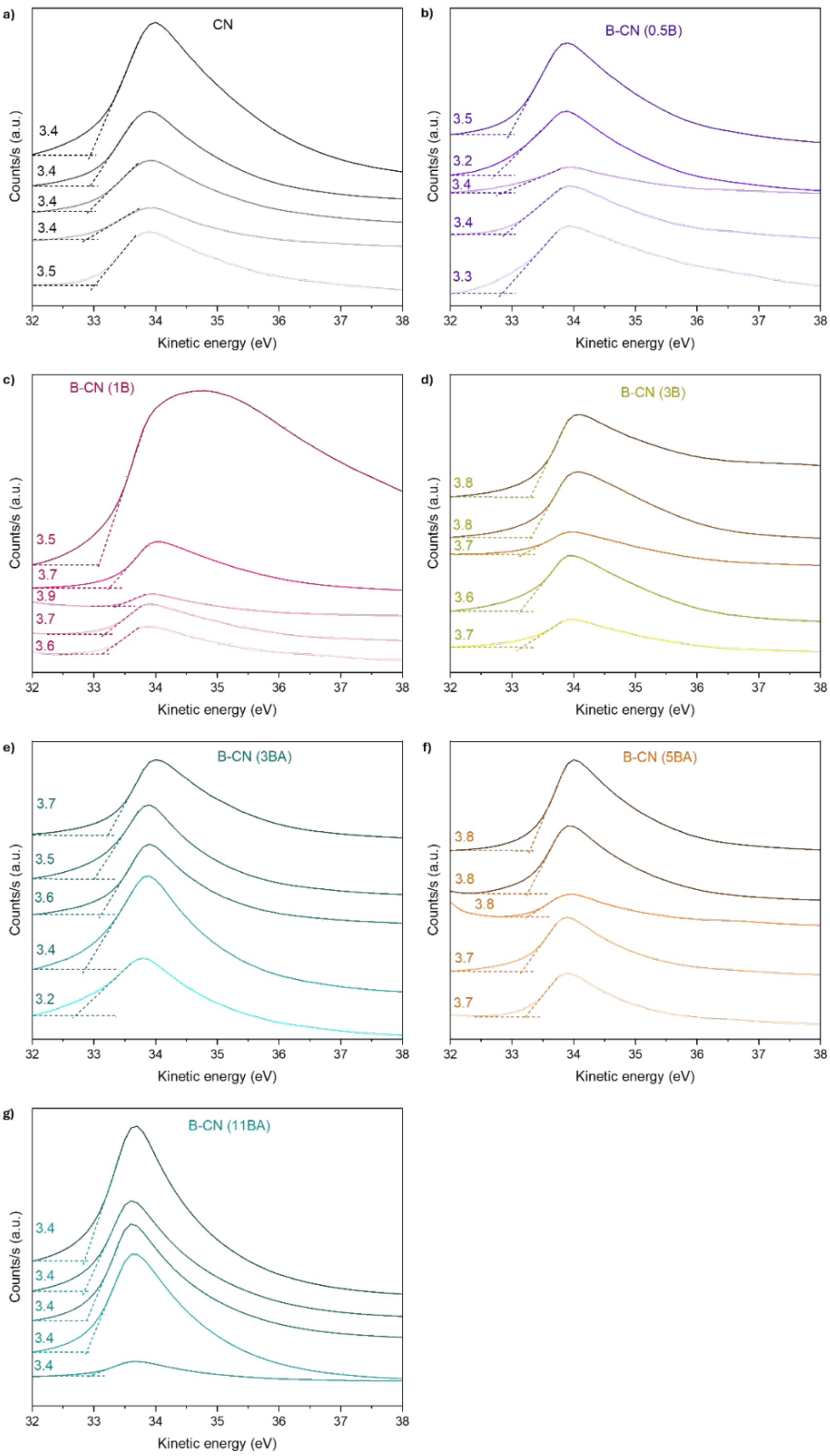

Figure S12. XPS work function plots for a) CN, b) B-CN (0.5B), c) B-CN (1B), d) B-CN (3B), e) B-CN (3BA), f) B-CN (5BA), and g) B-CN (11BA). The work function for each sample was calculated by averaging the five indicated values from the five different points.

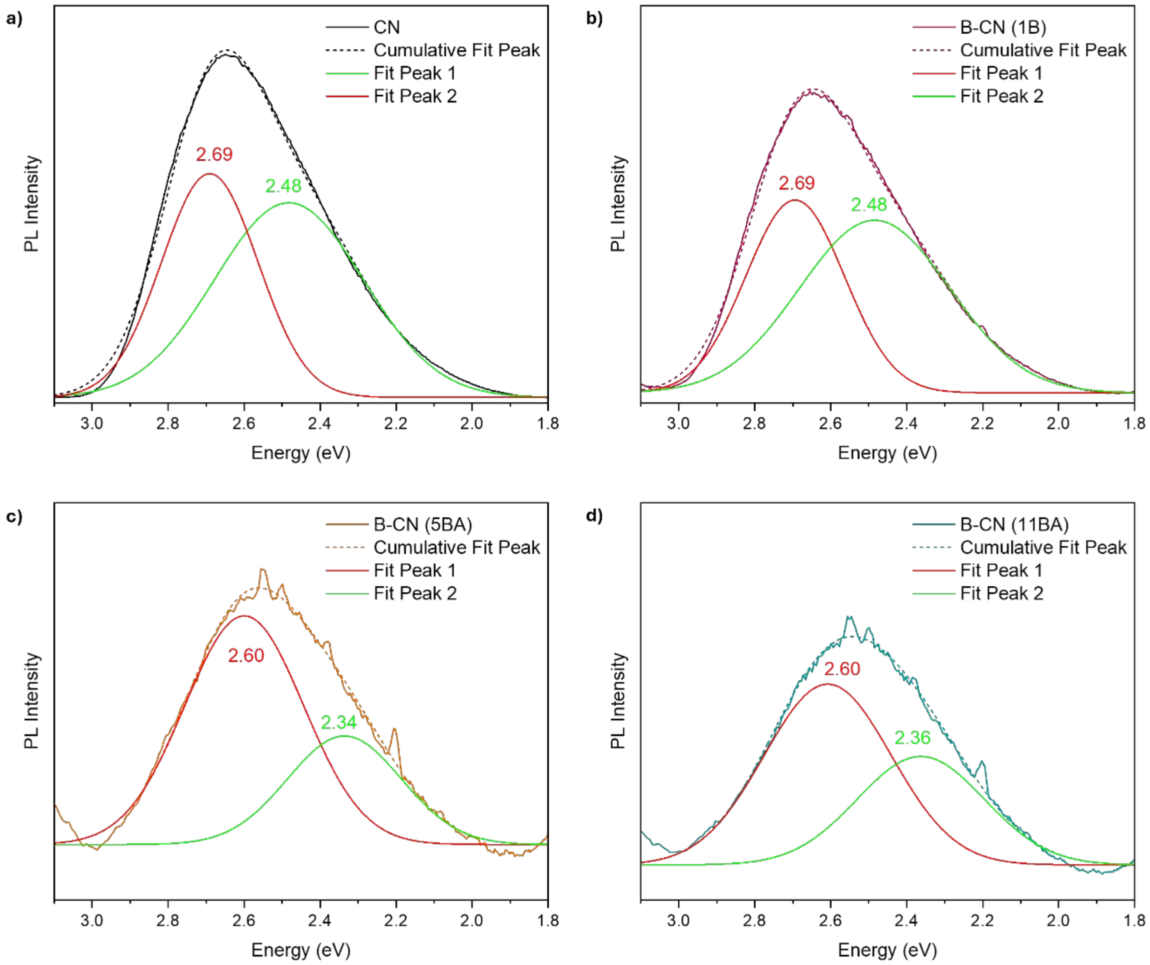

Figure S13. Steady-state PL data obtained upon excitation at 355 nm, fitted using the Gaussian fit for a) CN, b) B-CN (1B), c) B-CN (5BA), and d) B-CN (11BA). The main fitted peak for each sample can be deconvoluted into two peaks, presented as Fit Peak 1 and Fit Peak 2.

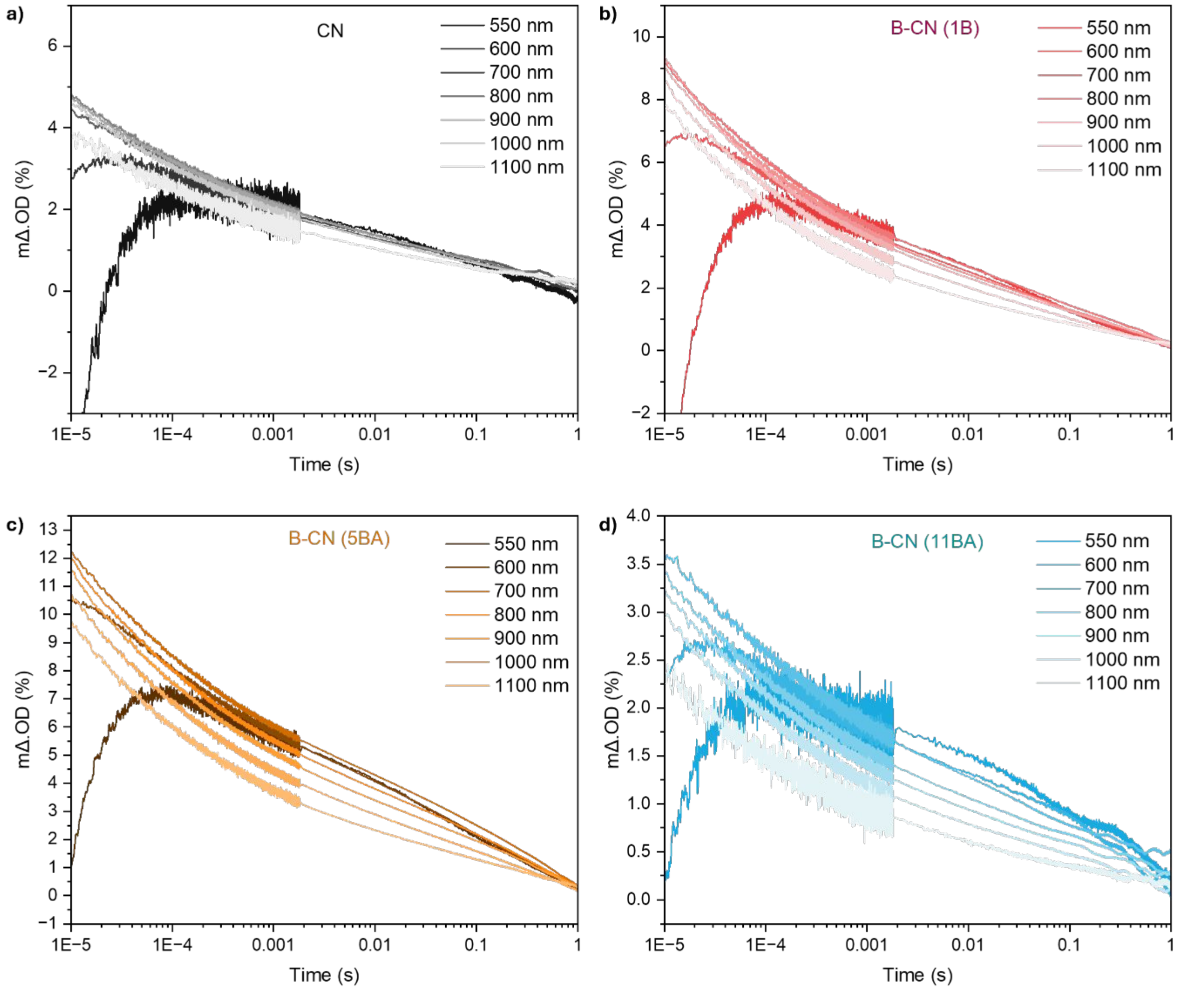

Figure S14. TAS decay profiles over time upon excitation at 355 nm, probed at 550-1100 nm for a) CN, b) B-CN (1B), c) B-CN (5BA), and B-CN (11BA).

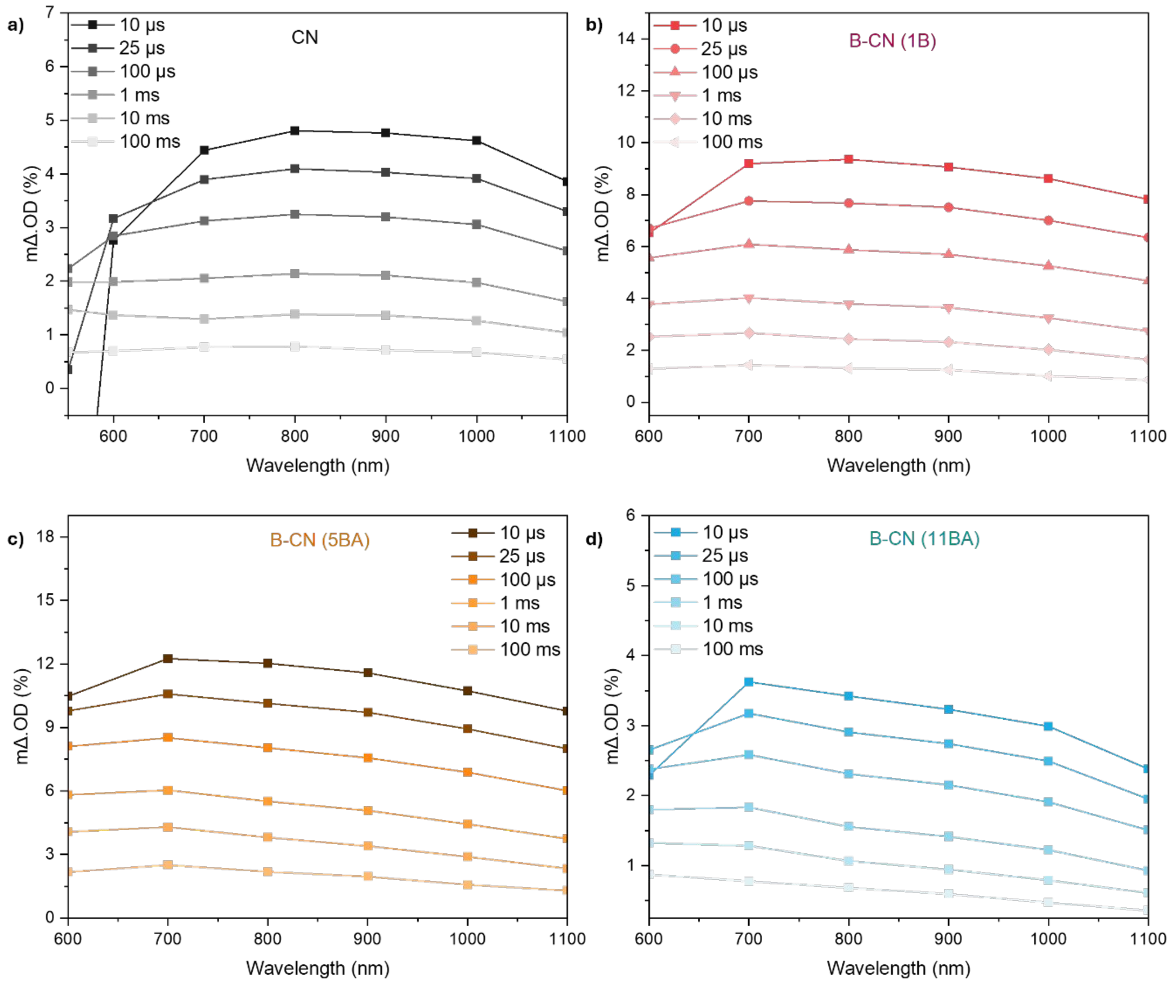

Figure S15. TAS data probed over 550-1100 nm, obtained upon excitation at 355 nm for a) CN, b) B-CN (1B), c) B-CN (5BA), and d) B-CN (11BA).

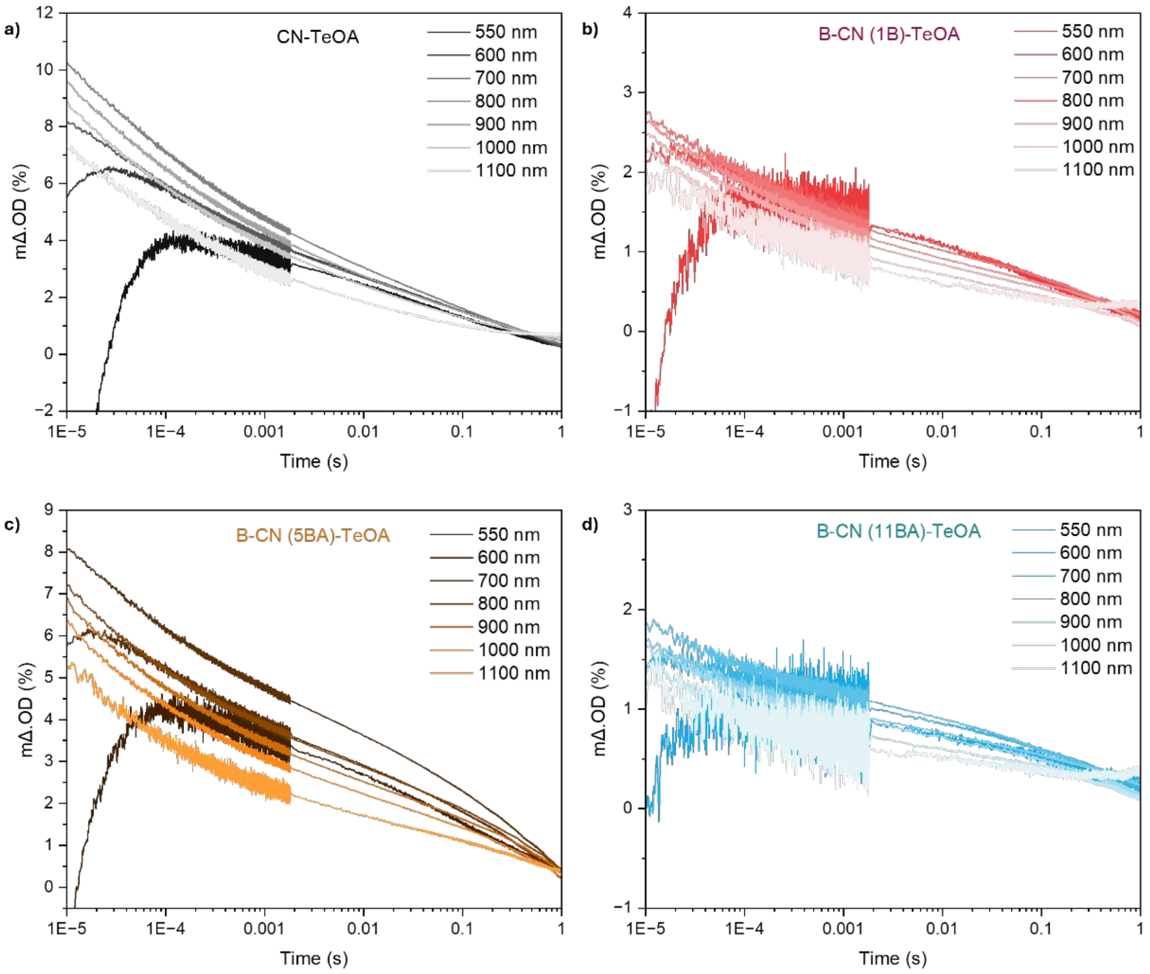

Figure S16. TAS decay profiles over time upon excitation at 355 nm, probed at 550-1100 nm for a) CN, b) B-CN (1B), c) B-CN (5BA), and B-CN (11BA), in the presence of the hole scavenger TeOA.

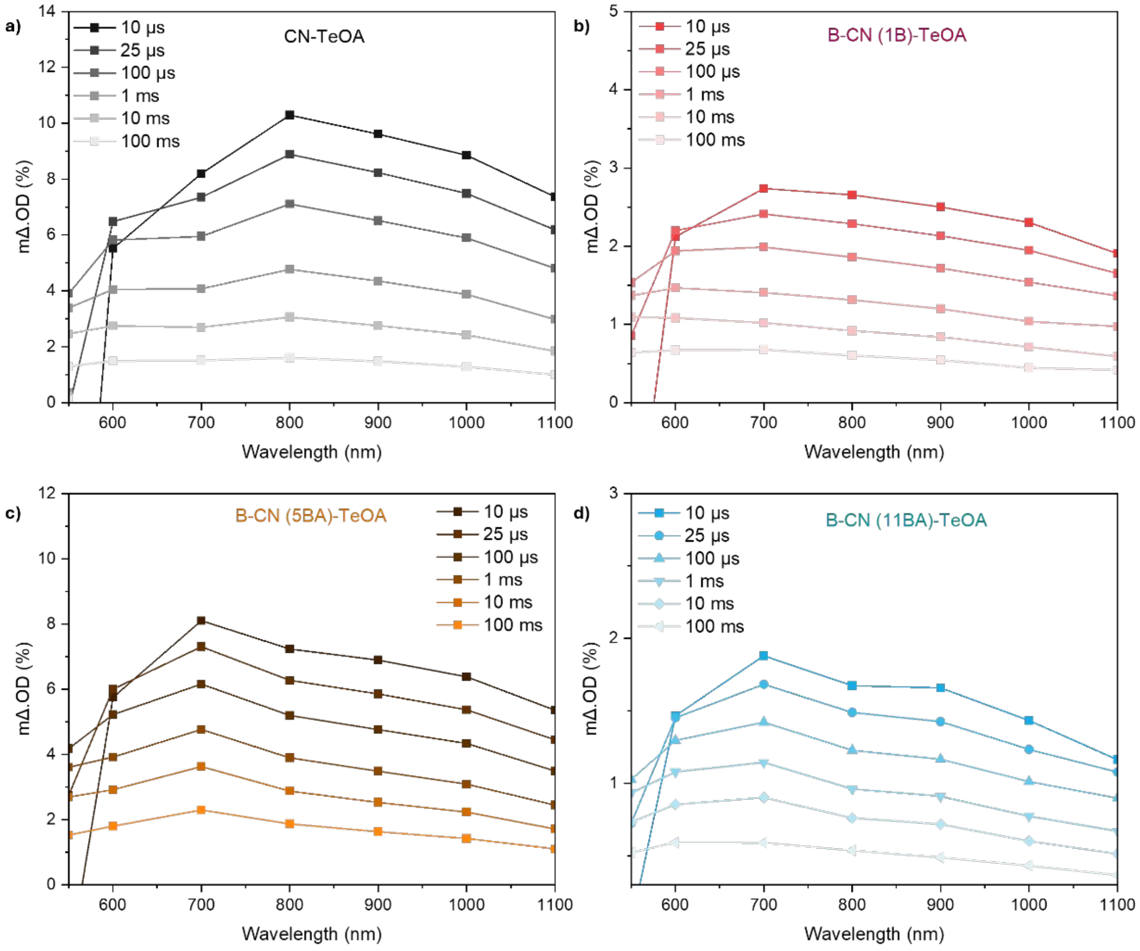

Figure S17. TAS data probed over 550-1100 nm, upon excitation at 355 nm for a) CN, b) B-CN (1B), c) B-CN (5BA), and B-CN (11BA), in the presence of the hole scavenger TeOA.

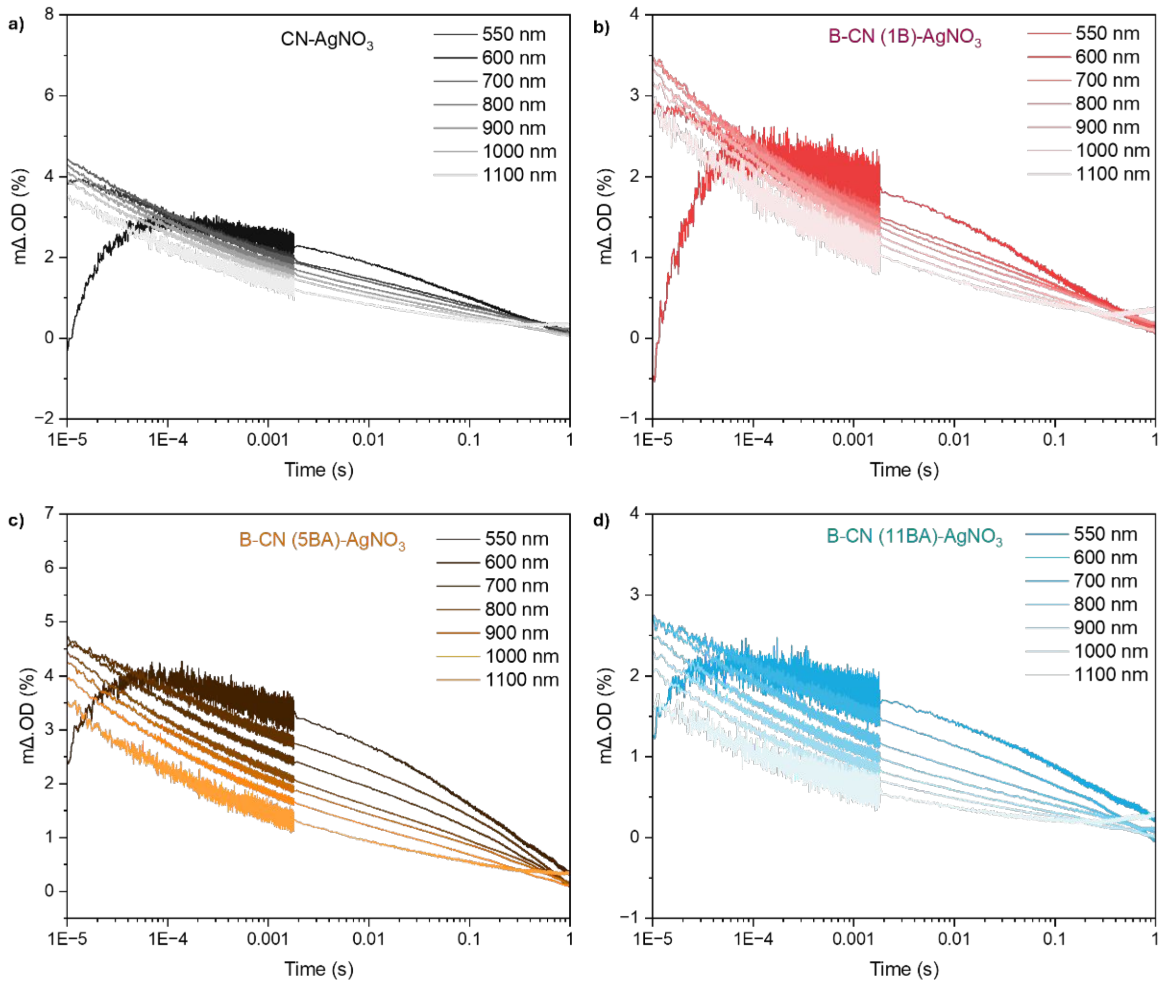

Figure S18. TAS decay profiles over time upon excitation at 355 nm, probed at 550-1100 nm for a) CN, b) B-CN (1B), c) B-CN (5BA), and B-CN (11BA), in the presence of the electron scavenger $AgNO_3$.

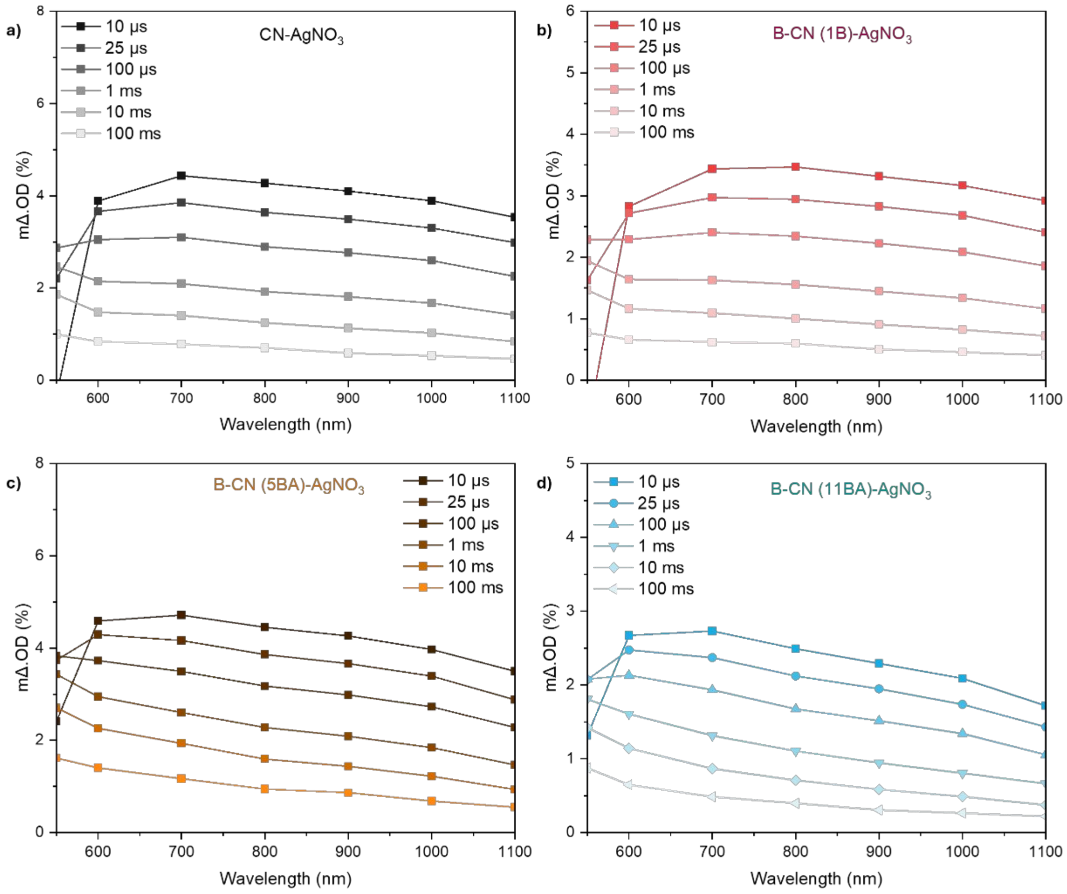

Figure S19. TAS kinetic data probed over 550-1100 nm, upon excitation at 355 nm for a) CN, b) B-CN (1B), c) B-CN (5BA), and B-CN (11BA), in the presence of the electron scavenger $AgNO_3$.

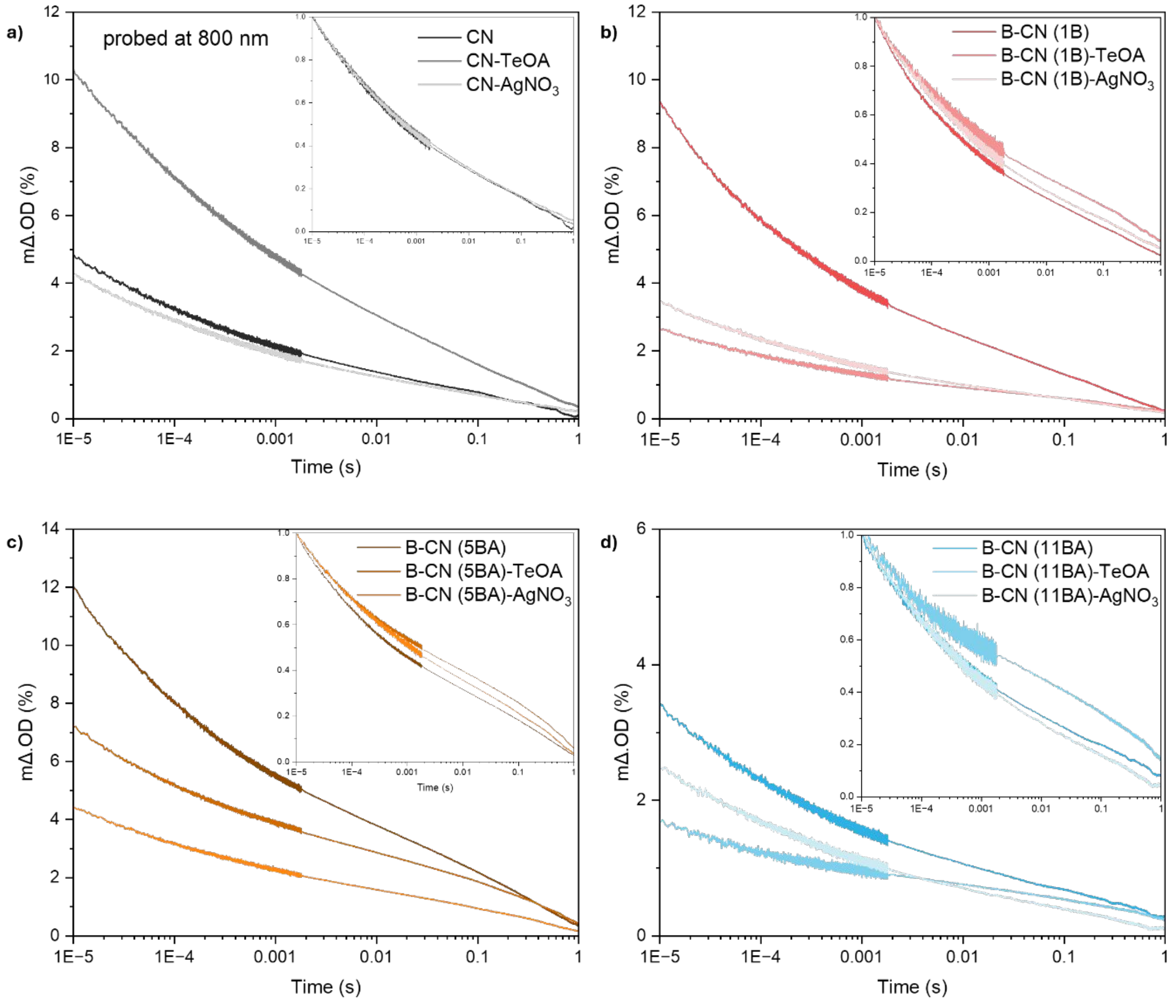

Figure S20. Comparison of TAS decay profiles over time upon excitation at 355 nm, probed at 800 nm for a) CN, b) B-CN (1B), c) B-CN (5BA), and B-CN (11BA), with and without use of the scavengers TeOA and $AgNO_3$. The inner plots present the normalised TAS decay profiles, under the same conditions.

Table S5. Comparison of photocatalytic activity between literature and current work: Operating conditions and performance of B-functionalized gC_3N_4 materials for the CO_2 photoreduction and NO_x photoremoval reactions.

Dopant	gC_3N_4 structure	Light source	Conditions	Proton donor/ Sacrificial agent	Product formed/ Conversion percentage	Ref
			**CO_2 PHOTOREDUCTION**			
B	bulk gC_3N_4	300 W Xe arc lamp	30 °C	H_2O	1 μmol g^{-1} h^{-1} CO<br>0.03 μmol g^{-1} h^{-1} CH_4<br>0.3 μmol g^{-1} h^{-1} H_2<br>(λ>420 nm)	21
B	gC_3N_4 nanosheets	300 W Xe arc lamp	Not reported	H_2O/H_2SO_4/ $NaHCO_3$	5 μmol g^{-1} h^{-1} CO<br>0.5 μmol g^{-1} h^{-1} CH_4<br>0.1 μmol g^{-1} h^{-1} H_2<br>3.8 μmol g^{-1} h^{-1} O_2<br>(λ=400 nm)	22
B	gC_3N_4 nanosheets	300 W Xe arc lamp	Not reported	H_2O	71 μmol g^{-1} h^{-1} CH_4<br>(λ>320 nm)<br>15-43 μmol g^{-1} h^{-1} CH_4<br>(λ>420 nm)	23
B	bulk gC_3N_4	300 W Xe arc lamp	Not reported	H_2O/H_2SO_4/ $NaHCO_3$	0.3-0.5 μmol g^{-1} h^{-1} CO<br>0.03-0.16 μmol g^{-1} h^{-1} CH_4<br>(λ>320 nm)	24
B, N, H	gC_3N_4 nanosheets	solar simulator	20-30 °C	H_2O	2-5 μmol g^{-1} h^{-1} CO<br>(λ>400 nm)	25
B	bulk gC_3N_4	solar simulator	25 °C	H_2O	5 μmol g^{-1} h^{-1} CO<br>(λ>400 nm)	26
B	ultrathin gC_3N_4 nanosheets	solar simulator	25 °C	H_2O	9 μmol g^{-1} h^{-1} CO<br>(λ>400 nm)	26
B, P	bulk gC_3N_4	solar simulator	25 °C	H_2O	11 μmol g^{-1} h^{-1} CO<br>(λ>400 nm)	26
B, P, N, H	ultrathin gC_3N_4 nanosheets	solar simulator	25 °C	H_2O	15-22 μmol g^{-1} h^{-1} CO<br>(λ>400 nm)	26
**B**	**bulk gC_3N_4**	**300 W Xe arc lamp<br>160 mW cm^{-2}**	**25 °C<br>1.7 bar**	**H_2O**	**2 μmol g^{-1} h^{-1} CO<br>2 μmol g^{-1} h^{-1} CH_4<br>(λ>320 nm)**	**This work**
			**NO_x PHOTOREMOVAL**			
B	gC_3N_4 hollow nanotubes	300 W Xe arc lamp<br>25.5 mW cm^{-2}	25 °C<br>400 ppb<br>NO/air	-	25-30% NO conversion<br>(λ>420 nm)<br>24-49% NO2 selectivity<br>(λ>420 nm)	27
B	bulk gC_3N_4	Xe arc lamp	400 ppb<br>NO/air	-	25-26% NO conversion<br>(λ>420 nm)	28
**B**	**bulk gC_3N_4**	**2×15 W lamps<br>1.5 mW cm^{-2}<br>2×15 W lamps<br>3.5 mW cm^{-2}**	**25 °C<br>3 ppm NO/air<br>50 % RH**	**-**	**7-14% NO conversion<br>(λ=352 nm)<br>< 2% NO conversion<br>(λ>400 nm)<br>2-7% NO_x conversion<br>(λ=352 nm)<br>< 1% NO_x conversion<br>(λ>400 nm)<br>26-65% NO_2 selectivity<br>(λ=352 nm)**	**This work**

References

(1) Frydman, L.; Harwood, J. S. Isotropic Spectra of Half-Integer Quadrupolar Spins from Bidimensional Magic-Angle Spinning NMR. *J. Am. Chem. Soc.* **1995**, *117* (19), 5367–5368. https://doi.org/10.1021/ja00124a023.

(2) Amoureux, J. P.; Fernandez, C.; Steuernagel, S. Z Filtering in MQMAS NMR. *J. Magn. Reson. - Ser. A* **1996**, *123* (1), 116–118. https://doi.org/10.1006/jmra.1996.0221.

(3) Pike, K. J.; Malde, R. P.; Ashbrook, S. E.; McManus, J.; Wimperis, S. Multiple-Quantum MAS NMR of Quadrupolar Nuclei. Do Five-, Seven- and Nine-Quantum Experiments Yield Higher Resolution than the Three-Quantum Experiment? *Solid State Nucl. Magn. Reson.* **2000**, *16* (3), 203–215. https://doi.org/10.1016/S0926-2040(00)00081-3.

(4) Fung, B. M.; Khitrin, A. K.; Ermolaev, K. An Improved Broadband Decoupling Sequence for Liquid Crystals and Solids. *J. Magn. Reson.* **2000**, *142* (1), 97–101. https://doi.org/10.1006/jmre.1999.1896.

(5) Brunauer, S.; Emmett, P. H.; Teller, E. Adsorption of Gases in Multimolecular Layers. *J. Am.Chem.Soc.* **1938**, *60* (2), 309–319.

(6) Dubinin, M. M.; Radushkevich, L. M. The Equation of the Characteristic Curve of Activated Charcoal. *Dokl. Akad. Nauk. SSSR* **1947**, *55*, 331–334.

(7) Džimbeg-malčić, V.; Barbarić-mikočević, Ž.; Itrić, K. Kubelka-Munk Theory in Describing Optical Properties of Paper (I). *Teh. Vjesn.* **2011**, *18* (1), 117–124.

(8) Džimbeg-malčić, V.; Barbarić-mikočević, Ž.; Itrić, K. Kubelka-Munk Theory in Describing Optical Properties of Paper (II). *Teh. Vjesn.* **2012**, *19* (1), 191–196.

(9) Tauc, J. Optical Properties and Electronic Structure of Amorphous Ge and Si. *Mater. Res. Bull.* **1968**, *3* (1), 37–46. https://doi.org/10.1016/0025-5408(68)90023-8.

(10) Wu, H. Z.; Liu, L. M.; Zhao, S. J. The Effect of Water on the Structural, Electronic and Photocatalytic Properties of Graphitic Carbon Nitride. *Phys. Chem. Chem. Phys.* **2014**, *16* (7), 3299–3304. https://doi.org/10.1039/c3cp54333a.

(11) Pickard, C. J.; Mauri, F. All-Electron Magnetic Response with Pseudopotentials: NMR Chemical Shifts. *Phys. Rev. B - Condens. Matter Mater. Phys.* **2001**, *63* (24), 2451011–2451013. https://doi.org/10.1103/physrevb.63.245101.

(12) Clark, S. J.; Segall, M. D.; Pickard, C. J.; Hasnip, P. J.; Probert, M. I. J.; Refson, K.; Payne, M. C. First Principles Methods Using CASTEP. *Zeitschrift fur Krist.* **2005**, *220* (5–6), 567–570. https://doi.org/10.1524/zkri.220.5.567.65075.

(13) Perdew, J. P.; Burke, K.; Ernzerhof, M. Generalized Gradient Approximation Made Simple. *Phys. Rev. Lett.* **1996**, *77* (18), 3865–3868. https://doi.org/10.1103/PhysRevLett.77.3865.

(14) Grimme, S.; Ehrlich, S.; Goerigk, L. Effect of the Damping Function in Dispersion Corrected Density Functional Theory. *J. Comput. Chem.* **2011**, *32* (7), 1456–1465. https://doi.org/10.1002/jcc.21759.

(15) Yates, J. R.; Pickard, C. J.; Payne, M. C.; Mauri, F. Relativistic Nuclear Magnetic Resonance Chemical Shifts of Heavy Nuclei with Pseudopotentials and the Zeroth-Order Regular Approximation. *J. Chem. Phys.* **2003**, *118* (13), 5746–5753. https://doi.org/10.1063/1.1541625.

(16) Monkhorst, H. J.; Pack, J. D. Special Points for Brillonin-Zone Integrations. *Phys. Rev. B* **1976**, *13* (12), 5188–5192. https://doi.org/10.1103/PhysRevB.16.1748.

(17) Gracia, J.; Kroll, P. Corrugated Layered Heptazine-Based Carbon Nitride: The Lowest Energy Modifications of C3N4 Ground State. *J. Mater. Chem.* **2009**, *19* (19), 3013. https://doi.org/10.1039/b821568e.

(18) Jürgens, B.; Irran, E.; Senker, J.; Kroll, P.; Müller, H.; Schnick, W. Melem (2,5,8-Triamino-Tri- s -Triazine), an Important Intermediate during Condensation of Melamine Rings to Graphitic Carbon Nitride: Synthesis, Structure Determination by X-Ray Powder Diffractometry, Solid-State NMR, and Theoretical Studies. *J. Am. Chem. Soc.* **2003**, *125* (34), 10288–10300. https://doi.org/10.1021/ja0357689.

(19) Jeschke, G.; Hoffbauer, W.; Jansen, M. A Comprehensive NMR Study of Cubic and Hexagonal Boron Nitride. *Solid State Nucl. Magn. Reson.* **1998**, *12* (1), 1–7. https://doi.org/10.1016/S0926-2040(98)00045-9.

(20) Pyykkö, P. Year-2017 Nuclear Quadrupole Moments. *Mol. Phys.* **2018**, *116* (10), 1328–1338. https://doi.org/10.1080/00268976.2018.1426131.

(21) Wang, H.; Tang, Q.; Wu, Z. Construction of Few-Layer Ti3C2 MXene and Boron-Doped g-C3N4 for Enhanced Photocatalytic CO2 Reduction. *ACS Sustain. Chem. Eng.* **2021**, *9* (25), 8425–8434. https://doi.org/10.1021/acssuschemeng.1c01155.

(22) Shi, X.; Zhang, Q.; Zhou, Y.; Ye, Q.; Jiang, D.; Tian, D.; Li, D. Boosting Charge Transfer in Au-Decorated B/K Co-Doped CN Nanosheets towards Enhanced Photocatalytic CO2 Reduction. *Mater. Chem. Front.* **2023**, *7* (10), 2049–2058. https://doi.org/10.1039/d3qm00056g.

(23) Raziq, F.; Qu, Y.; Zhang, X.; Humayun, M.; Wu, J.; Zada, A.; Yu, H.; Sun, X.; Jing, L. Enhanced Cocatalyst-Free Visible-Light Activities for Photocatalytic Fuel Production of g-C3N4 by Trapping Holes and Transferring Electrons. *J. Phys. Chem. C* **2016**, *120* (1), 98–107. https://doi.org/10.1021/acs.jpcc.5b10313.

(24) Fu, J.; Liu, K.; Jiang, K.; Li, H.; An, P.; Li, W.; Zhang, N.; Li, H.; Xu, X.; Zhou, H.; et al. Graphitic Carbon Nitride with Dopant Induced Charge Localization for Enhanced Photoreduction of CO2 to CH4. *Adv. Sci.* **2019**, *6* (18), 1900796. https://doi.org/10.1002/advs.201900796.

(25) Hussien, M. K.; Sabbah, A.; Qorbani, M.; Hammad Elsayed, M.; Raghunath, P.; Lin, T.

Y.; Quadir, S.; Wang, H. Y.; Wu, H. L.; Tzou, D. L. M.; et al. Metal-Free Four-in-One Modification of g-C3N4 for Superior Photocatalytic CO2 Reduction and H2 Evolution. *Chem. Eng. J.* **2022**, *430* (P2), 132853. https://doi.org/10.1016/j.cej.2021.132853.

(26) Hussien, M. K.; Sabbah, A.; Qorbani, M.; Putikam, R.; Kholimatussadiah, S.; Tzou, D. L. M.; Elsayed, M. H.; Lu, Y. J.; Wang, Y. Y.; Lee, X. H.; et al. Constructing B─N─P Bonds in Ultrathin Holey G-C3N4 for Regulating the Local Chemical Environment in Photocatalytic CO2 Reduction to CO. *Small* **2024**, *20* (35), 1–14. https://doi.org/10.1002/smll.202400724.

(27) Wang, Z.; Chen, M.; Huang, Y.; Shi, X.; Zhang, Y.; Huang, T.; Cao, J.; Ho, W.; Lee, S. C. Self-Assembly Synthesis of Boron-Doped Graphitic Carbon Nitride Hollow Tubes for Enhanced Photocatalytic NOx Removal under Visible Light. *Appl. Catal. B Environ.* **2018**, *239* (June), 352–361. https://doi.org/10.1016/j.apcatb.2018.08.030.

(28) Jin, X.; Guan, Q.; Tian, T.; Li, H.; Han, Y.; Hao, F.; Cui, Y.; Li, W.; Zhu, Y.; Zhang, Y. In2O3/Boron Doped g-C3N4 Heterojunction Catalysts with Remarkably Enhanced Visible-Light Photocatalytic Efficiencies. *Appl. Surf. Sci.* **2020**, *504* (November 2019), 144241. https://doi.org/10.1016/j.apsusc.2019.144241.
